# Local High-Dose Radiotherapy Induces Systemic Immunomodulating Effects of Potential Therapeutic Relevance in Oligometastatic Breast Cancer

**DOI:** 10.3389/fimmu.2017.01476

**Published:** 2017-11-06

**Authors:** Elena Muraro, Carlo Furlan, Michele Avanzo, Debora Martorelli, Elisa Comaro, Aurora Rizzo, Damiana A. Fae’, Massimiliano Berretta, Loredana Militello, Alessandro Del Conte, Simon Spazzapan, Riccardo Dolcetti, Marco Trovo’

**Affiliations:** ^1^Immunopathology and Biomarker Unit, Department of Translational Research, CRO Aviano National Cancer Institute, Aviano, Italy; ^2^Department of Radiation Oncology, CRO Aviano National Cancer Institute, Aviano, Italy; ^3^Division of Medical Physics, CRO Aviano National Cancer Institute, Aviano, Italy; ^4^Department of Medical Oncology, CRO Aviano National Cancer Institute, Aviano, Italy; ^5^Department of Medical Oncology, Pordenone General Hospital, Aviano, Italy; ^6^Translational Research Institute, University of Queensland Diamantina Institute, Brisbane, QLD, Australia; ^7^Department of Radiation Oncology, Azienda Sanitaria Universitaria Integrata of Udine, Udine, Italy

**Keywords:** oligometastatic breast cancer, stereotactic body radiotherapy, antitumor T cell responses, immunomodulation, radiotherapy immunogenicity

## Abstract

Local irradiation of cancer through radiotherapy can induce spontaneous regression of non-directly irradiated lesions, suggesting the involvement of systemic antitumor immune responses. In oligometastatic breast cancer (BC) patients, the use of stereotactic body radiotherapy (SBRT) favors the local control of treated lesions and may contribute to break local tolerance and release tumor-associated antigens (TAAs), improving host antitumor immunity. We performed a detailed immunomonitoring of BC patients undergoing SBRT to verify its ability to “switch on” the anti-tumor immunity both systemically, in peripheral blood, and locally, employing *in vitro* BC models. Twenty-one BC patients with ≤6 metastases were treated with 3 daily doses of 10 Gy with SBRT. Blood samples for immune profiling were collected before and after treatment. One month after treatment a third of patients displayed the boosting or even the *de novo* appearance of polyfunctional CD4^+^ and CD8^+^ T cell responses against known BC TAAs (survivin, mammaglobin-A, HER2), through intracellular staining in flow cytometry. Half of patients showed increased numbers of activated natural killer (NK) cells, measured with multispectral flow cytometry, immediately after the first dose of SBRT. Interestingly, high levels of activated NK cells at diagnosis correlated with a longer progression-free survival. BC *in vitro* models, treated with the same SBRT modality, showed enhanced expression of MHC class-I and class-II, major histocompatibility complex class I-related chain A/B, and Fas molecules, and increased release of pro-inflammatory cytokines, such as IL-1β and TNF-α. Consistently, we noticed enhanced production of perforin by CD4^+^ T cells when patients’ lymphocytes were cultured in the presence of irradiated BC cell line, compared to untreated targets. Besides immunogenic effects, SBRT also enhanced the percentages of circulating regulatory T cells, and increased *indoleamine 2,3 dioxygenase* and PD-L1 expression in BC *in vitro* models. These results suggest that SBRT may boost host antitumor immune responses also in an advanced disease setting such as oligometastatic BC, by inducing immunomodulating effects both locally and systemically. However, the concomitant induction of immunosuppressive pathways suggests that a combination with immunotherapy could further enhance the *in situ* vaccination ability of radiotherapy, possibly further improving the curative potential of SBRT in this subset of patients.

## Introduction

The 10- to 20-year relapse-free survival observed in 2% of metastatic breast cancer (BC) indicates that a small proportion of metastatic patients can achieve prolonged survival (1). In this setting, particular interest is aroused by the oligometastatic state, namely the presence of ≤6 metastases before tumor cells acquire widespread metastatic potential (2), a clinical condition observed in about 1–10% of newly diagnosed patients with metastatic BC (2, 3), that can occur also after cytoreductive or curative locoregional therapy (4). The relatively limited extension of oligometastasis suggests that radiotherapy (RT) targeting solitary lesions, alone or in combination with systemic therapy, could potentially cure these patients (1). In this context, delivery of few RT fractions with large (10 Gy or more) fractional doses (5) through stereotactic body radiotherapy (SBRT) may enhance local control and improve progression-free survival (PFS) and overall survival (6). However, the identification of reliable predictive markers able to identify patients who could benefit from this treatment is still a challenge (4). Compared to conventional RT, SBRT may mediate different biological effects including (i) induction of vascular and stromal changes together with direct cell killing within the high-dose region; (ii) activation of host innate and adaptive immunity, thus contributing to the local and systemic tumor control (2). Therefore, we wondered whether the characterization of host immunity during SBRT treatment could provide biomarkers potentially useful to identify those patients who could mostly benefit from this therapy.

Interestingly, spontaneous tumor regression at sites distant to the irradiated volume (abscopal effect) has been documented after RT (7), at a median response time of 6 months. The delayed timing of abscopal effects suggests the possible activation of a cascade of antitumor immune responses (7). These effects are more frequent in patients with a vigorous immune system and, when combined with immune modulating drugs, RT seems to drive a “vaccine effect,” able to sensitize host immune system to recognize previously unnoticed tumor cells (8). The ablative high-dose per fractionation of SBRT may imply an even greater enhancement of antitumor immune responses than conventional RT (9). Indeed, high doses of RT induce cell apoptosis/necrosis and generate an inflammatory environment in which dendritic cells may engulf dying tumor cells/apoptotic bodies and effectively present tumor antigens to T lymphocytes, thereby boosting antitumor immunity. Tumor-specific T cells activated locally may then circulate and recognize metastatic lesions, favoring the abscopal effects induced by RT (10). Moreover, at sub-lethal doses, radiation is able to induce immunogenic effects, including upregulation of MHC class I and adhesion molecules, such as ICAM-1, on surviving tumor cells, which are then more susceptible to recognition and killing by T cells (10, 11).

The ability to induce immunogenic effects is particularly relevant in BC, which was long considered as a poorly immunogenic cancer, given its low prevalence in the immunosuppressed population (12). Nevertheless, the immune system seems to play a pivotal role in preventing BC metastases (13) and the extent of lymphocytic infiltration is considered a good prognostic indicator in BC, with several differences depending on molecular subtype (14). Interestingly, the prognostic role of intratumor immune cell infiltration may reflect also at the systemic level, as we recently demonstrated by showing improved natural killer (NK) activity, retained tumor-specific T-cell responses, and unaltered cytokine levels in BC patients undergoing pathological complete response compared to those achieving only partial responses (15, 16).

On these grounds, monitoring immune responses in blood may allow the identification of biomarkers potentially indicative of the “switching-on” of antitumor immunity triggered by RT, and to follow its evolution along the treatment (8). Moreover, the characterization of tumor-specific T-cell responses and cytokine induction could be used to optimize the antitumor effects of radiation protocols (7), particularly when RT is combined with chemotherapy and HER2-targeting antibodies, whose responses are markedly influenced by immune-related factors (13, 17).

At our best knowledge, the immunogenicity of SBRT has still not been characterized in the oligometastatic BC, a condition that could imply a widespread immune suppression, which could still be reverted when employing immunogenic therapies. The aim of the present study was thus to measure the immunogenic effects of SBRT in BC, both at a systemic level, investigating blood samples from oligometastatic BC patients before and after SBRT, and locally, employing BC *in vitro* models, due to unreachable patients tumor tissues. Finally, we wondered whether SBRT could favor the tumor cell recognition by T lymphocytes. We interestingly detected the induction of immunogenic effects in a significant fraction of patients treated with SBRT, including the improvement of antitumor T-cell responses and the upregulation of MHC molecules on cancer cells. However, these effects were paralleled by the concomitant engagement of immunosuppressive pathways, such as PD-L1/PD-1, which could hinder the antitumor activity of RT mediated by its ability to “switch on” patients’ immune system. The proved immunomodulation induced systemically by SBRT prompted us to evaluate the potential predictive role of cytokines, immune cells, and antitumor responses to identify potential biomarkers of PFS, easily detectable in the liquid biopsy.

## Materials and Methods

### Patients Assessments and Therapy

Immunomodulating effects of SBRT were measured in peripheral blood samples obtained from 21 oligometastatic BC patients (median age 53, range 41–87) enrolled in a phase 2 prospective clinical trial, between January 2012 and December 2015. Inclusion criteria were as follows: metastatic BC with ≤6 metastases; extent of disease assessed with FDG-PET/CT and, in case of liver metastases, also with a MRI of the abdomen; Eastern Cooperative Oncology Group performance status <2; primary tumor controlled; absence of brain metastasis. The use of concomitant systemic therapies, such as hormonal- or chemotherapy, steroids, and trastuzumab, was allowed. Radiotherapeutic treatment was delivered using SBRT technique, which consisted in 30 Gy in three fractions, to all metastatic sites. The diameter of metastatic lesions treated with SBRT was between about 5 and 20 mm. Primary clinical end-point was PFS at 2 years from the end of SBRT treatment. We included also 14 age-matched healthy women as controls. The study was conducted with the approval of the local institutional review board. Written informed consent was obtained from all patients and donors. All subjects gave written informed consent in accordance with the Declaration of Helsinki.

### Sample Collection

Blood and serum samples were collected from patients before treatment, 24 h after the first dose of SBRT, 1, and 4 months after RT treatment, and transported at room temperature. Peripheral blood mononuclear cells (PBMCs) were freshly isolated (within 5 h after blood drawing) from heparinized blood of patients by Ficoll-Hypaque gradient (Lymphoprep, Fresenius Kabi Norge Halden) using standard gradient separation. Cells were washed in PBS (Biomerieux), counted using Trypan blue (viability >90%) and viably frozen [90% heat-inactivated fetal bovine serum (FBS; Euroclone) and 10% DMSO] at −80°C for 24 h and then in liquid nitrogen until use. After thawing in IMDM (Lonza) containing 2 mM l-glutamine, 100 µg/ml streptomycin, and 100 IU/ml penicillin (Sigma-Aldrich), supplemented with 2% human serum (Sigma-Aldrich) and with 3 µg/ml deoxyribonuclease (Sigma-Aldrich), cells were washed in PBS (Biomerieux) and counted again to check viability (>80%). Serum samples were obtained with blood centrifugation at 890 *g* and maintained at −80°C. Healthy donors PBMCs were collected from buffy coat products and stored as described above. Patient and donor samples were genotyped to identify those expressing the alleles HLA-A*0201, -A*0301, -A*2402, -B*3501, -DRB1*0101, -DRB1*0301, -DRB1*0401, and -DRB1*1501 by performing PCR sequencing based typing with specific primers (18, 19). HLA background is reported in Table S1 in Supplementary Material.

### Culture Conditions and Treatment of BC Cell Lines and Primary T Cells

Because the irradiated tumor tissue of oligometastatic BC patients was unreachable for *in situ* analysis, we performed parallel *in vitro* experiments on BC models. The following BC cell lines were employed in this study: the HER2-overexpressing MDA-MB453, the luminal type MCF7, and the basal type MDA-MB231. Each cell line was authenticated by fingerprinting in November 2015 (GenePrint^®^ 10 System, Promega, Madison, WI, USA) and HLA genotyping was performed as described above (MDA-MB453 expresses HLA-DRB1*03; MCF7 expresses HLA-A*02 and -DRB1*03; MDA-231 expresses HLA-A*02 and -DRB1*03). All cell lines were cultured in DMEM (Sigma), containing 2 mM l-glutamine, 10% FCS (Gibco^®^, Life Technology, Grand Island, NY, USA), 100 µg/ml streptomycin, and 100 IU/ml penicillin (Sigma-Aldrich, St. Louis, MO, USA), at 37°C in 5% of CO_2_.

Cells were γ-irradiated using a ^137^Cs irradiator (Nordion) with doses of 10 or 30 Gy. To perform *in vitro* irradiation with the same prescribed dose, fractionation, and beam energy used in SBRT for BC patients, cell plates were irradiated with dose of 10 Gy of 6-MV photons delivered by a linear accelerator (Varian Clinac 600C, Varian Medical System, Palo Alto, CA, USA) for three consecutive days, 24 h apart. During the irradiation, cell plates were positioned between two 5 cm layers of solid water, with LINAC gantry at 180°. The dose was checked by radiochromic film dosimetry in the same setup of irradiation. Seventy-two hours after treatment, cell culture supernatant was collected and maintained at −80°C; cells were counted using Trypan blue and compared to untreated controls before proceeding with further analyses.

Spontaneous T-cell responses against known BC-associated antigens were evaluated in prestimulated patients’ PBMCs (20). Briefly, thawed PBMCs were cultured in T-cell medium (IMDM containing 2 mM l-glutamine, 100 µg/ml streptomycin, and 100 IU/ml penicillin, supplemented with 10% human serum) in the presence of 5 ng/ml IL-7 (PromoKine) and IL-4 (PromoKine), at 37°C and 7.5% of CO_2_. One day after thawing, cells were stimulated with 1 µg/ml HLA-matched peptides, from BC-associated antigens (survivin, mammaglobin-A, and Her2/neu) or viral controls (HIV, Flu, CMV, EBV), supplemented with 5 ng/ml IL-7 and IL-4, and cultured at 37°C and 7.5% CO_2_. Selected epitopes with the corresponding source, HLA restriction, and reference are listed in Table S2 in Supplementary Material. Cells were supplemented with 2 ng/ml IL-2 (PromoKine) every 2 days. After 12 days, prestimulated cells were collected, counted, and stimulated overnight with individual peptides, in the presence of α-CD107a FITC (mouse IgG1, H4A3; BD Biosciences), Golgi-STOP solution (protein transport inhibitor containing monensin, BD Biosciences), and 10 µg/ml Brefeldin (Sigma-Aldrich). Non-specific stimulation with 5 ng/ml phorbol 12-myristate 13-acetate (PMA, Sigma-Aldrich) and 1 µg/ml ionomycin (Sigma-Aldrich) was employed as control for cytokine production.

To verify the different ability of T cells to recognize BC cell lines, we co-cultured patients’ PBMCs, collected before and after SBRT, with untreated or irradiated HLA-matched BC cell lines. Untreated or irradiated BC cell lines were collected 72 h after irradiation, washed, and labeled with carboxyfluorescein diacetate succinimidyl ester (CFSE; Molecular Probes). Briefly, BC cell lines (3 × 10^6^/ml) were stained in PBS with 2 µM CFSE for 10 min at room temperature; an equal volume of FBS was added for 20 min at room temperature and finally cells were washed four times with IMDM supplemented with FCS. BC cells were counted again and co-cultured with PBMCs at an effector:target ratio of 2:1 in T-cell medium in the presence of α–CD107a Pe (mouse IgG1, H4A3; BD Biosciences), Golgi-STOP solution, and 10 µg/ml Brefeldin overnight at 37°C and 7.5% of CO_2_.

### Flow Cytometry and Multispectral Imaging

The antibodies used for flow cytometry and multispectral imaging are listed in Table S3 in Supplementary Material. DRAQ5™ fluorescent DNA dye from BioStatus Limited was used in nuclear localization analysis; LIVE/DEAD^®^ Fixable Aqua Dead Cell Stain (Molecular Probes, Thermo Fisher Scientific) was used to determine cell viability. Properly labeled isotypic antibodies were used as negative controls. All antibodies were used in an appropriate volume of 2% FCS and PBS to reduce non-specific signal and re-suspended in an appropriate volume of 1% paraformaldehyde in PBS. Intracellular FoxP3 and Ki-67 were determined using the eBioscience FoxP3 Staining Buffer Set (eBioscience), according to the manufacturer’s instructions. Briefly, after surface molecules staining, cells were fixed and permeabilized with fixation/permeabilization buffer for 30 min at 4°C, washed twice, and labeled with FoxP3 and Ki-67 antibodies in the presence of permeabilization buffer at 4°C for at least 30 min, and after two washes, cells were re-suspended in PBS. To characterize T helper 17 cells, PBMCs were pretreated with 50 ng/ml PMA (Sigma-Aldrich) and 1 µg/ml ionomycin (Sigma-Aldrich) in the presence of Golgi-STOP solution (protein transport inhibitor containing monensin, BD Biosciences) and 10 µg/ml Brefeldin (Sigma-Aldrich) in T cell medium for 4 h at 37°C. To evaluate IL-17, IL-22, TNF-α, IFN-γ, IL-2, MIP-1β, and perforin production, cells were labeled for surface molecules, then fixed and permeabilized with the Cytofix/Cytoperm™ solution (BD Biosciences) for 20 min at 4°C, washed in PBS with 0.5% bovine serum albumin (BSA; Sigma-Aldrich) and 0.1% saponin (Sigma-Aldrich), and stained with antibodies in PBS + BSA + saponin at 4°C for 20 min. Samples were washed twice and re-suspended in PBS for flow cytometry analysis. For PBMCs analysis, at least 2 × 10^5^ and 5 × 10^5^ events were acquired, respectively, for surface markers and intracellular staining, while 1 × 10^4^ events were collected for cell lines evaluations. Flow cytometry analysis was performed with a Cytomics FC500 (Beckman Colter, Fullerton, CA, USA), and a LSR-Fortessa™ (Becton Dickinson) belonging to the flow cytometry core facility of our Institute. Photomultiplier voltages and compensation were set with unstained and stained cells or with the CompBeads Set Anti-Mouse Ig or Anti-Rat Ig, k Sets (BD Biosciences). Flow cytometry data were analyzed with CXP (Beckman Colter, Fullerton, CA, USA), DIVA (BD) and FlowJo (Tree Star, Ashland, OR, USA) software; Boolean gating analysis for intracellular staining was performed with Pestle and Spice software. The production of cytokines after stimulation with tumor-associated antigens was considered positive if at least doubled compared to the percentage of cytokine-positive cells after stimulation with negative controls (HIV-derived peptides; Figure S1 in Supplementary Material).

To determine NF-κB nuclear internalization, 2 × 10^6^ PBMCs were labeled with α-NF-κB FITC and α-CD56 PE monoclonal antibodies. Briefly, after CD56 staining in 10% rabbit serum (Dako), cells were fixed with 2% of paraformaldehyde in complete medium [RPMI-1640 (Sigma-Aldrich) supplemented with 10% FBS] for 10 min at room temperature. Cells were washed once in complete medium, permeabilized in cold methanol 90% in PBS, and incubated 10 min in ice; thereafter, cells were washed twice and labeled with NF-κB antibody in 2% rabbit serum (Dako) in PBS/0.5% BSA (Sigma-Aldrich) for 30 min at 4°C. After two washes in PBS/0.5% BSA, cells were re-suspended in PBS with 1% paraformaldehyde and DRAQ5 DNA dye. Cells were run on the ImageStreamX cytometer using the INSPIRE software (Amnis Corporation, Seattle, WA, USA). For each sample, at least 10^4^ CD56-positive events were acquired. Cells were excited using a 488 nm laser with intensity of 40 mW. Brightfield, side scatter, fluorescent cell images were acquired at 40× magnification. Only events with brightfield areas greater than 20 µm^2^ (excluding debris) and non-saturating pixels were collected. Data were analyzed with the IDEAS software (Amnis Corporation, Seattle, WA, USA); NF-κB nuclear localization was measured through the Similarity Score (SS) feature, which defines/quantifies the similarity of the nuclear (DRAQ5) and labeled transcription factor staining patterns (NF-κB FITC). All events showing a positive SS were considered with high similarity between NF-κB and DRAQ5, thus indicating a nuclear localization of the transcription factor. Only viable cells were selected on the basis of morphologic features. Single-stained compensation controls were used to compensate fluorescence between channel images on a pixel-by-pixel basis.

### Antibody-Dependent Cell Cytotoxicity (ADCC) Assay

The monoclonal antibody trastuzumab acts through widely described immune-mediated mechanisms as the ADCC which involves host’s immune cells (21). The ADCC efficiency was evaluated in a Calcein-AM release assay, using the HER2/neu-overexpressing BC cell line MDA-MB453 as target cells, and patients PBMCs as effectors. Target cells (one million) in exponential growth were labeled with 7.5 µM Calcein-AM (Molecular Probes) for 30 min at 37°C, washed three times, and then incubated with trastuzumab antibody (20 µg/ml; Roche) 1 h in ice. Without washing the persistence of soluble antibody, 1 × 10^4^-labeled target cells per well were seeded into 96-well U-bottom plates. Experiments were conducted in triplicates at two effector (PBMCs):target ratios of 30:1 and 15:1, in 200 µl of Hank’s balanced salt solution (HBSS) containing 5% FCS. After 4 h at 37°C and 5% CO2, the release of Calcein (excitation = 485 nm; emission = 530 nm) was measured with a fluorescence plate reader (SpectraFluor Plus, Tecan, Männedorf, Switzerland). Maximal and spontaneous Calcein release values were obtained by adding either 100 µl lysis buffer (NaBO_3_ 0.025 M, Triton X-100 0.1%, pH 9) or HBSS, to wells containing 1 × 10^4^ labeled target cells. The percentage of lysis was calculated according to the standard formula = 100 × (experimental release − spontaneous release)/(maximal release − spontaneous release).

### RNA Extraction, cDNA Synthesis, and Quantitative Real-time PCR

After 72 h from irradiation, 1.5/2 × 10^6^ cells were collected and washed twice in PBS. Total RNA was isolated by QIAGEN RNeasy Mini Kit (QIAGEN). One microgram of RNA was retro-transcribed into cDNA using the Iscript RT OneTube Supermix (BIO-RAD, Hercules, CA, USA) according to manufacturer’s instructions. *Indoleamine 2,3 dioxygenase* (*IDO*) primers (Sigma-Aldrich), forward GCTGGTGGAGGACATGCTGCT and reverse ACCAGAGCTTTCACACAGGCGT, were designed with Primer3 (version 0.4.0) and sequence specificity control was performed by BLAST alignment tool. Primer sequences for the reference gene *HPRT* were kindly provided by BIO-RAD. Quantitative real-time PCR analysis were performed in a CFX96 Thermal Cycler, using SsoFast EvaGreen Supermix (BIO-RAD, Hercules, CA, USA). Normalized fold expression was calculated with the formula 2^−ΔΔCq^, using the Bio-Rad CFX Manager software.

### Multiplex ELISA: Ciraplex^®^ and Luminex

Serum levels of interleukin IL-1β, IL-6, IL-8, IL-10, and TNF-α were evaluated using the Ciraplex^®^ multiplex arrays (Aushon BioSystems, TEMA Ricerca, Bologna, Italy), according to the manufacturer’s instructions. Briefly, custom human 5-plex array with pre-spotted cytokine-specific antibodies was used. Standards and pre-diluted samples were added in duplicate and, after 1 h of incubation at room temperature and three washes, biotinylated antibody reagent was added to each well. After 30 min incubation at room temperature and three washes, block solution was added to stabilize the signal. The addition of Streptavidin-HRP Reagent and SuperSignal^®^ Substrate, and the acquisition of luminescent signal with a cooled Charge Coupled Device camera, together with data analysis and processing, were performed by TEMA Ricerca laboratories’ customer service (Bologna, Italy).

Cell culture supernatants were evaluated through Bio-Plex Pro™ Human Chemokine Assay to quantify the levels of IL-1β, IL-6, IL-8, IL-10, TNF-α, CXCL9 (MIG), CXCL10 (IP10), and CXCL16, 72 h after γ-radiation or fractionated RT. Experiments were carried out under manufacturer’s instructions. Briefly, antibodies-coupled beads were seeded in all wells of a 96-well plate, washed twice, and incubated with standards or properly diluted samples in duplicate for 1-h shaking. After three washes, detection antibodies were added to each well and incubated for 30 min shaking. After washing again three times, we added streptavidin-PE for 10 min, washed three times, and respuspended beads in proper buffer to finally read the plate on the Luminex200 (Luminex Corporation, Austin, TX, USA). The XPonent software package (version 3.1) was used to calculate sample cytokine concentrations, expressed as picograms per milliliter.

### Statistical Analysis

Data obtained from multiple independent experiments were expressed as mean and SD for immunophenotypic analysis and *in vitro* experiments on BC models (mean and SD of three independent experiments); as median and quartiles for cytokine serum data. The Student’s *t*-test for two-tailed distributions and paired data was used for the statistical analysis of variations before and after SBRT in circulating immune cells percentages and for the statistical analysis of protein expression and cytokine release in *in vitro* BC models irradiated or not treated. The Student’s *t*-test for two-tailed distributions and unpaired data was used to compare immunophenotype data regarding oligometastatic BC patients with those obtained from healthy women. The Wilcoxon signed-rank test was used for the statistical analysis of variation in cytokines serum data before and after SBRT in oligometastatic BC patients, and the Mann–Whitney test was employed to compare cytokine serum levels of BC patients with those of healthy women. The Student’s *t*-test for two-tailed distributions and unpaired data was used to compare the baseline immune parameters levels of relapsing patients, with those obtained from not-relapsing patients. For correlative analysis patient population was divided into two groups for each immune parameter [IL-6, IL-8, IL-10, nuclear translocation of NF-κB in NK cells, B cells, T lymphocytes, regulatory T cells (Treg), and T helper 17 cells] using the marker baseline median value as cutoff point and identifying “high” and “low” subgroups. PFS was defined as the time from the end of treatment to local or distant progression, or death from any cause. PFS was estimated using the Kaplan–Meier method, starting from the end of RT to the event of interest or last available follow-up. The log-rank test (two-sided) was used to test the differences between the subgroups. In all cases, statistical significance was considered for *p* < 0.05.

## Results

### Patients Characteristics

Globally, 21 patients were included in this translational study. Patients’ characteristics are listed in Table 1. Median age was 51 (37–84); 16 patients had luminal BC; 4 Her2-positive BC; and 1 triple negative BC. All patients except two had ductal carcinoma. The oligometastatic status was induced by previous treatments in 11/21 patients: 8 patients had 1 metastatic lesion, 10 patients had 2 lesions, 1 patient had 3 lesions, 1 patient had 4 lesions, and 1 patient had 6 lesions. Metastatic localization was mainly bone (16/21 patients), lymph node in 3 patients, 1 patient had both bone and lymph node localization, and 1 presented a metastatic lesion in the lung. Patients with HER2-positive BC received concomitant trastuzumab, 1 patient was concomitantly treated with chemotherapy, 15/21 patients received hormonal therapy. Local control, defined as “absence of local progression,” was achieved in all patients, while systemic progression was documented in 13/21 patients. The main localization of metastatic progression was bone (8/13), followed by lymph node (2/13), brain (1/13), liver (1/13), and lung (1/13). No abscopal responses were documented in any patient.

**Table 1:** Patient characteristics.

Patient number	Age	Type of BC	Histology	ER PgR	Grade	Oligometastatic status	Number of lesions	Metastatic localization	RT fractions	Systemic treatment	Systemic progression after RT	Site of progression	PFS (months after SBRT)
1	47	Luminal	Ductal	ER^+^PgR^+^	2	Induced	6	Bone	3	Hormonal	Yes	Bone	14
3	70	Luminal	Ductal	ER^+^PgR^+^	3	*De novo*	3	Bone and lymph nodes	3	Chemotherapy	Yes	Brain	18
4	57	Luminal	Ductal	ER^−^PgR^+^	3	*De novo*	2	Bone	3	No	No		30
5	43	HER2	Ductal	ER^+^PgR^+^	3	Induced	2	Bone	3	Trastuzumab	Yes	Bone	14
6	37	Luminal	Ductal	ER^+^PgR^−^	2	*De novo*	1	Bone	3	Hormonal	No		27
7	39	HER2	Ductal	ER^−^PgR^+^	3	Induced	2	Bone	3	Trastuzumab	No		41
8	49	Luminal	Ductal	ER^+^PgR^−^	3	*De novo*	2	Bone	3	Hormonal	Yes	Liver	22
9	84	Luminal	Ductal	ER^+^PgR^+^	3	*De novo*	1	Lung	3	Hormonal	Yes	Lymph node	18
10	81	Luminal	Ductal	ER^+^PgR^+^	Unk	*De novo*	2	Bone	3	Hormonal	Yes	Bone	13
11	51	Luminal	Ductal	ER^+^PgR^+^	2	*De novo*	1	Bone	3	Hormonal	Yes	Bone	11
12	51	Luminal	Lobular	ER^+^PgR^+^	2	Induced	2	Bone	3	Hormonal	Yes	Bone	6
13	52	Luminal	Ductal	ER^+^PgR^+^	3	*De novo*	1	Lymph node	3	Hormonal	Yes	Lymph node	13
14	58	HER2	Ductal	ER^−^PgR^−^	2	Induced	1	Bone	3	Trastuzumab	Yes	Lung	16
15	40	Luminal	Ductal	ER^+^PgR^+^	3	Induced	2	Bone	3	Hormonal	Yes	Bone	4
16	81	Luminal	Ductal	ER^+^PgR^+^	3	Induced	2	Lymph node	3	Hormonal	No		28
17	47	HER2	Ductal	ER^+^PgR^+^	3	*De novo*	2	Bone	3	Hormonal + trastuzumab	No		24
19	39	TNBC	Ductal	ER^−^PgR^−^	3	Induced	4	Lymph node	3	No	No		21
20	54	Luminal	Ductal	ER^+^PgR^+^	3	Induced	2	Bone	3	Hormonal	Yes	Bone	4
21	52	Luminal	Ductal	ER^+^PgR^+^	3	Induced	1	Bone	3	Hormonal	No		19
22	57	Luminal	Ductal	ER^+^PgR^+^	2	Induced	1	Bone	3	Hormonal	Yes	Bone	3
23	44	Luminal	Lobular	ER^+^PgR^+^	2	*De novo*	1	Bone	3	Hormonal	No		13

*BC, breast cancer; ER, estrogen receptor; PgR, progesterone receptor; RT, radiotherapy; PFS, progression-free survival; HER2, breast cancer overexpressing human epidermal growth factor receptor 2; TNBC, triple negative breast cancer; unk, unknown*.

### Increased T Cell Responses against BC-Associated Antigens after SBRT

**Figure 1:**
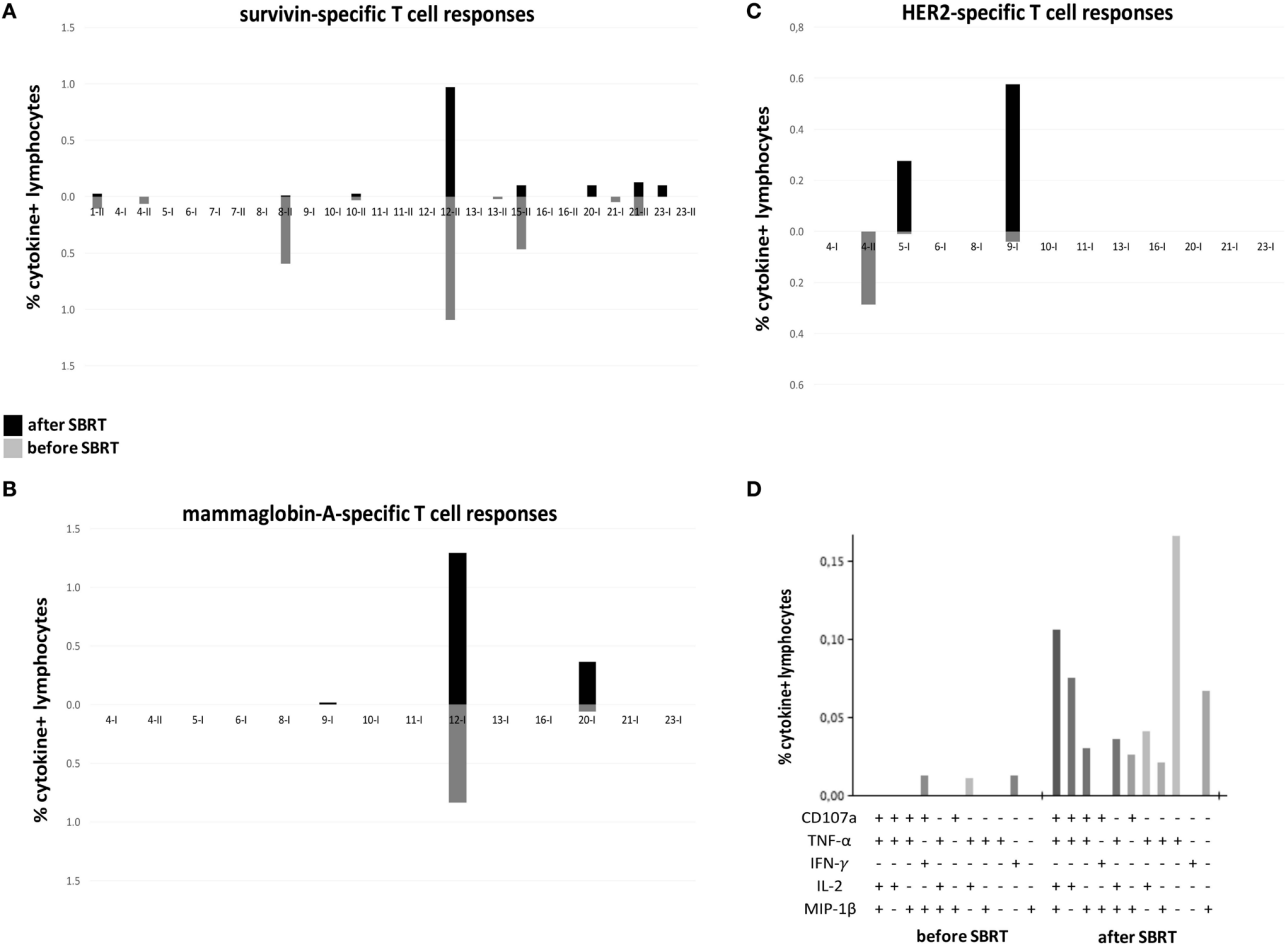
Tumor-specific T cell responses in oligometastatic breast cancer (BC) patients before and after SBRT. T cell responses against BC-associated antigens [survivin **(A)**; mammaglobin-A **(B)**; HER2 **(C)**] were monitored through flow cytometry in oligometastatic BC patients before and after (1 month; 4 months for patients no. 5 and no. 8) SBRT. The percentage of cytokine^+^ T cells was considered positive if at least doubled compared to the percentage of cytokine^+^ T cells after stimulation with a negative peptide control (HIV). Graphs in panels **(A–C)** reported the sum of T-cells positive to at least one of the markers investigated (CD107a, TNF-α, INF-γ, IL-2, and MIP-1β). A cutoff of 0.01% was set up to discriminate a positive population. Each pair of histograms (gray, data before SBRT; black, data after SBRT) represents the analysis performed in a single patient, as reported below, after stimulation with MHC class-I (I)- or MHC class-II (II)-restricted peptides. **(D)** Representative histogram of Boolean analysis performed in patient no. 9 and showing polyfunctional T cells after stimulation with MHC class-I-restricted HER2-derived peptides, before and after SBRT. Each bar represents a population of T cells producing a distinct set of cytokines or expressing CD107a as reported below.

To evaluate the potential immunogenic modulation of high doses RT and the consequent “vaccine-like” effect of local irradiation, we monitored the presence and the amount of circulating CD4^+^ and CD8^+^ T cell responses against known epitopes derived from the BC-associated antigens survivin, mammaglobin-A, and HER2/neu before and after RT, globally in 16 patients (due to availability of biological material collected 1 month after RT) enrolled in this study (exemplary flow cytometry dot plots are shown in Figure S1 in Supplementary Material). In 11/16 patients, we found a positive signal to at least one of the aforementioned antigens (patients no. 1, 4, 8, 10, 12, 13, 15, and 21 showed survivin-specific T cells, patients no. 12 and 20 mammaglobin-specific T cells, patients no. 4, 5, and 9 HER2-specific T cells), already before RT treatment, thus confirming the presence of spontaneous T-cell responses in BC patients elicited by the tumor itself (Figures 1A–C; Figure S2 in Supplementary Material). Interestingly, in 5/16 patients, we registered an increase (mammaglobin-specific T-cells in patients no. 12 and 20, HER2-specific T cells in patients no. 5 and 9) or even the *de novo* appearance (survivin-specific T cells in patients no. 20 and 23, mammaglobin-specific T cells in patient no. 9) of antitumor T-cell responses after SBRT (Figures 1A–C; Figure S2 in Supplementary Material), strongly suggesting a direct role of RT in promoting or boosting antitumor immune responses. Furthermore, after SBRT, BC antigen-specific T cells also displayed polyfunctional activity, as shown by the production of a broader set of cytokines per cell as compared to pre-RT levels (Figure 1D).

### Monitoring of NK Cells Activity

**Figure 2:**
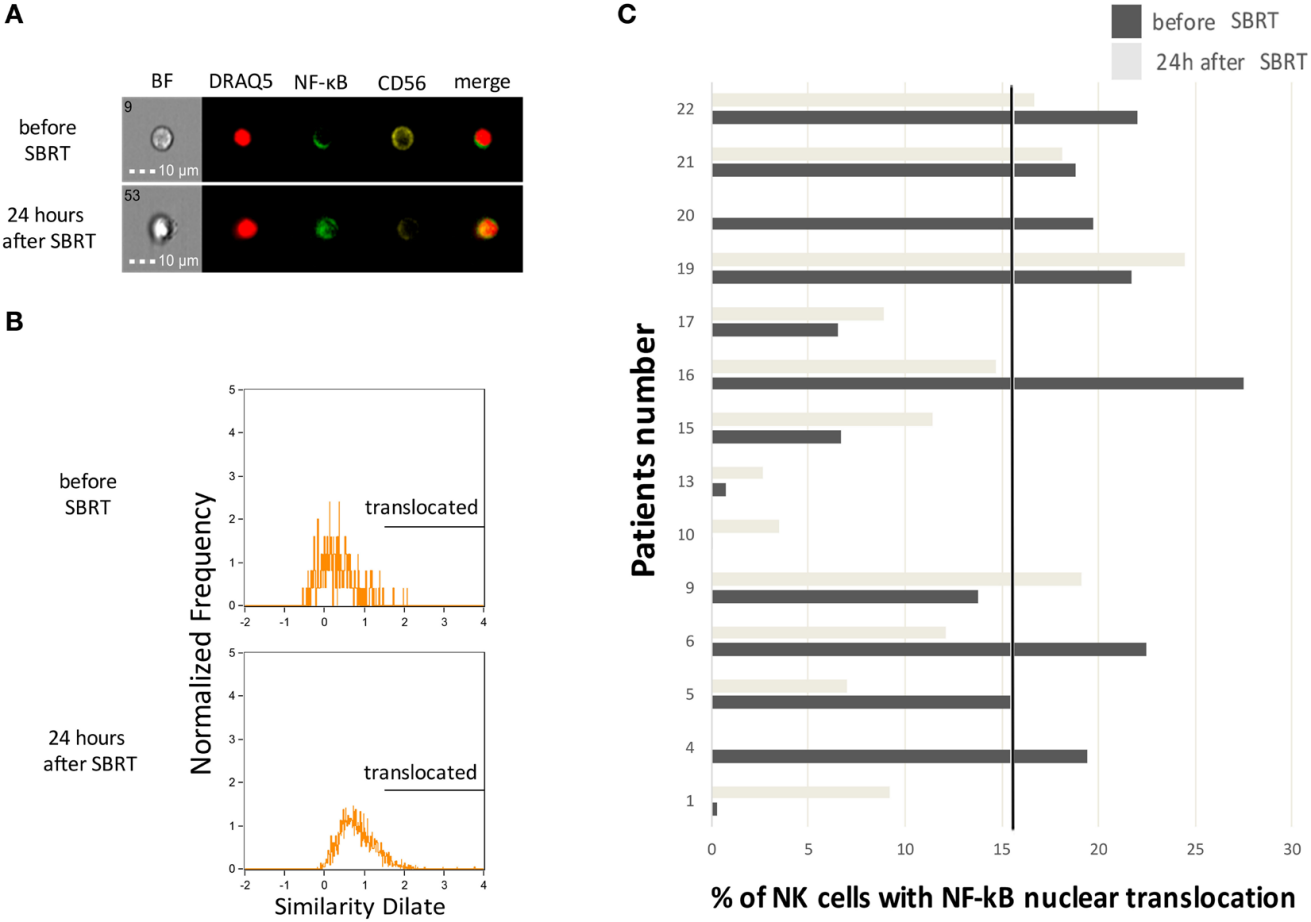
Natural killer (NK) cell activation status before and after SBRT. **(A)** Representative cell pictures obtained by multispectral flow cytometry on cell samples from patient no. 10 before and 24 h after the first SBRT fraction. BF, brightfield. **(B)** Representative multispectral flow cytometry plots showing typical histograms of NK cells without (upper plot) or with (lower plot) the nuclear translocation of NF-κB. Analysis performed on cell samples obtained from patient no. 10 before and 24 h after the first SBRT fraction. **(C)** Percentage of NK cells showing nuclear translocation of the p65 subunit of the NF-κB complex measured through multispectral flow cytometry, in patients before and 24 h after SBRT. In patients no. 4 and 20, the analysis was performed only before SBRT due to sample availability. The solid dot line represents the baseline median value.

Natural killer cells have a critical role in antitumor immunity and in particular they contribute to the induction of clinical responses when RT is associated with drugs acting through immune-mediated mechanisms, such as trastuzumab (22). We thus investigated circulating NK cells for the activation of the NF-κB transcription factor through multispectral flow cytometry, which allows a precise enumeration of cells carrying a nuclear translocation of the NF-κB p65 protein, as a marker of NF-κB activation (Figures 2A,B). Globally, we monitored NK cells obtained from 14 patients at diagnosis and 12 (due to availability of biological material collected 24 h after RT) of them also 24 h after the first dose of SBRT. We observed increased numbers of NK cells with nuclear translocation of NF-κB in 7/12 patients shortly (24 h) after the first dose of SBRT (Figure 2C), consistent with a rapid activation of NK cells in more than half of patients. We also quantified the percentages of perforin^+^ cells within NK cells in seven patients, but we did not observe evident variations (Figure S3 in Supplementary Material).

In parallel, we performed an *in vitro* ADCC assay for those patients treated with concomitant trastuzumab (*n* = 2). Although the analysis was carried out in a limited number of patients, our results are consistent with a direct correlation between the extent of NF-κB nuclear translocation in NK cells immediately after the first dose of SBRT and the percentage of trastuzumab-dependent ADCC lysis (Figure S4 in Supplementary Material), suggesting that RT may directly contribute to the functional activity of trastuzumab through modulation of NK cell activity.

### Immunophenotyping of Circulating Immune Cells

**Figure 3:**
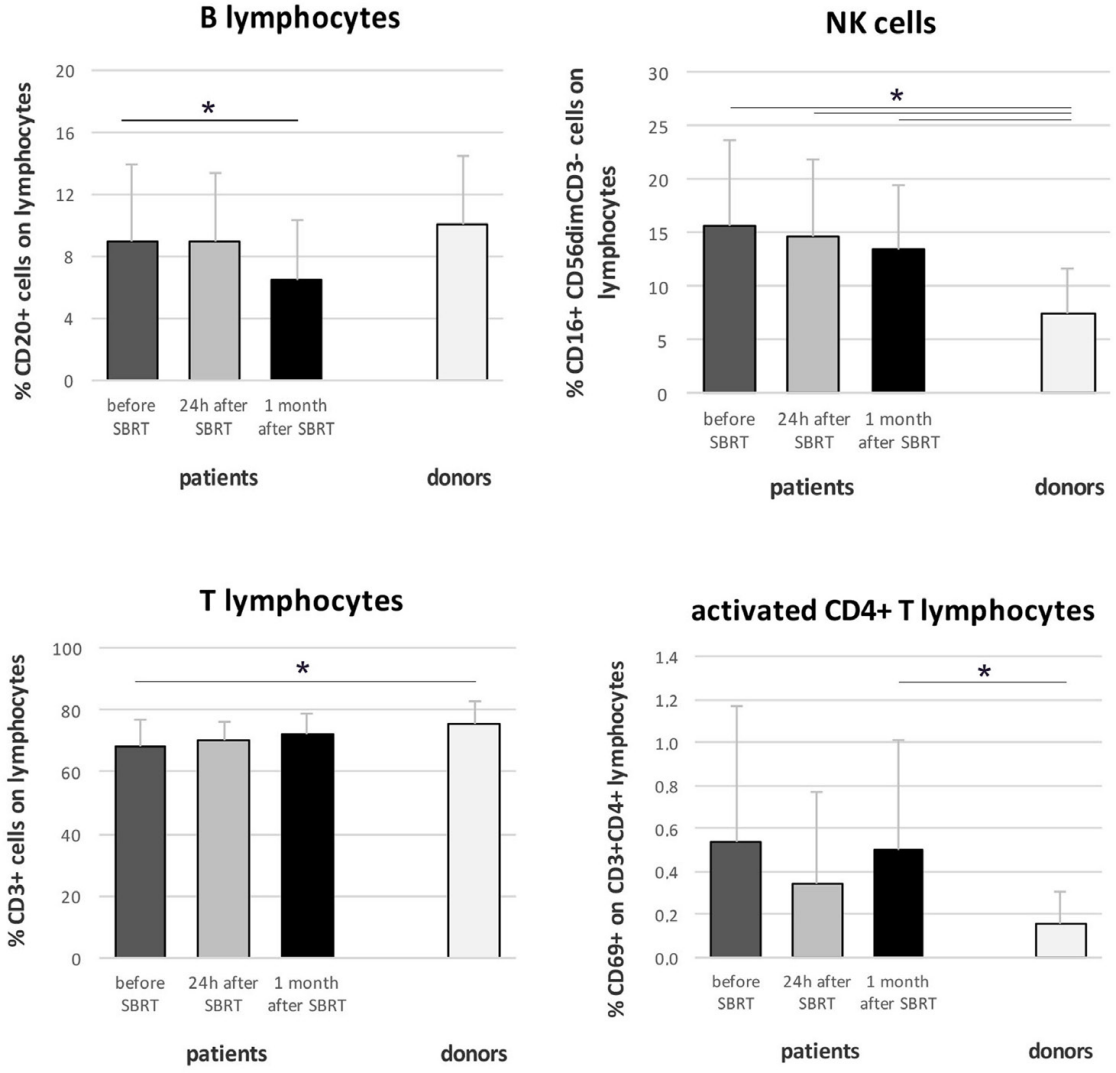

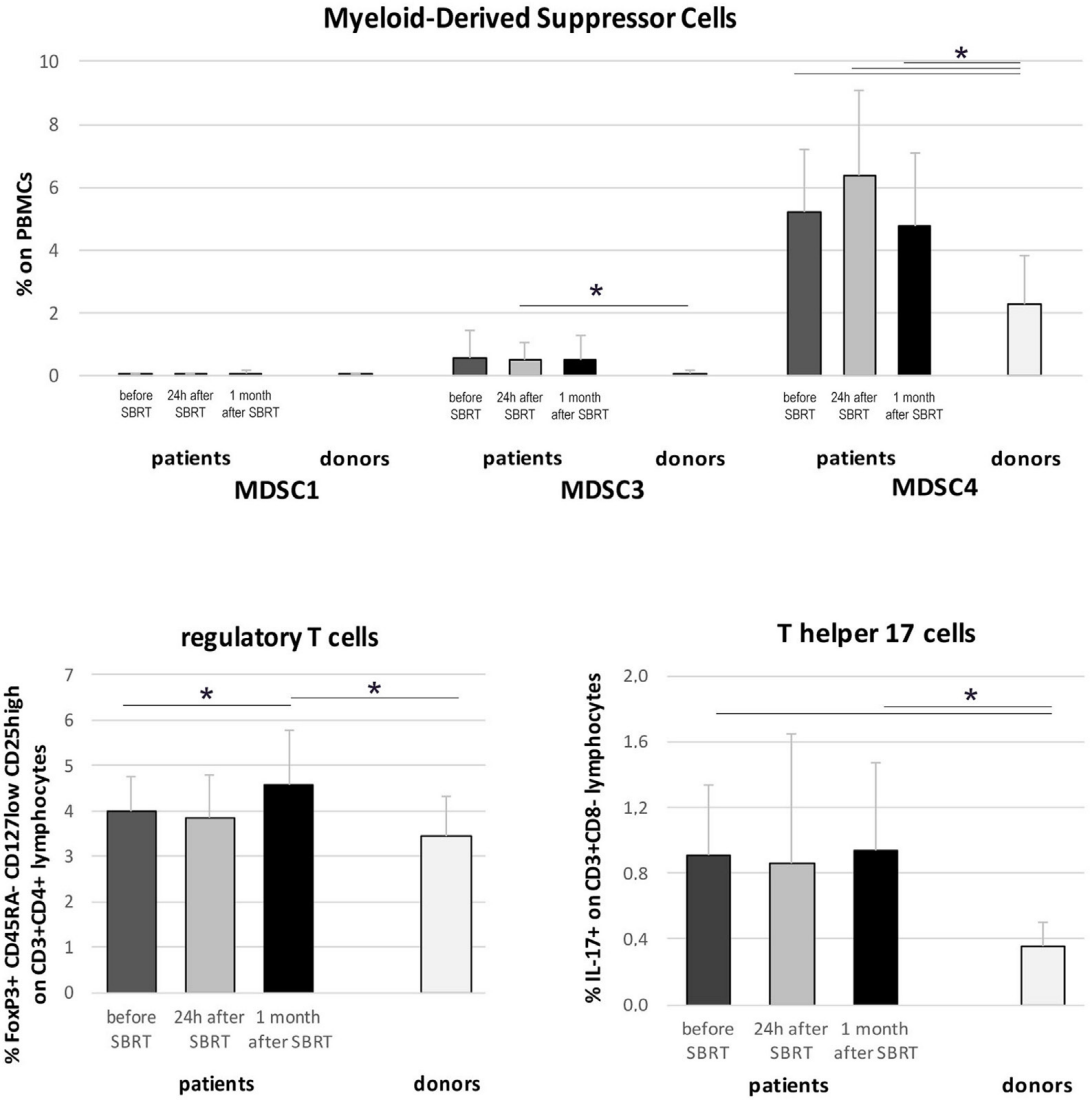
Immunophenotyping in oligometastatic breast cancer (BC) patients before and after SBRT. Flow cytometry characterization of immune populations in the peripheral blood of oligometastatic BC patients (*n* = 12) before (dark gray), 24 h after the first SBRT fraction (light gray), and 1 month after SBRT (black) and in the peripheral blood of healthy women (donors; *n* = 9). Cell subsets were identified as follows: B cells as CD20^+^ lymphocytes, natural killer (NK) cells as CD3^−^ CD16^+^ CD56^dim^ lymphocytes, T cells as CD3^+^ lymphocytes, activated CD4^+^ cells as CD3^+^ CD4^+^ CD69^+^ lymphocytes, MDSC1 as CD14^+^ CD124^+^ cells, MDSC3 as CD33^+^ Lineage^−^ HLA-DR^−^ cells, MDSC4 as CD14^+^ HLA-DR^−^ SSCim cells, regulatory T cells as CD3^+^ CD4^+^ CD25^high^ CD127^low^ CD45RA^−^ FoxP3^+^ lymphocytes, and T helper 17 cells as CD3^+^ CD8^−^ IL-17^+^ lymphocytes (*p ≤ 0.05).

Immunophenotyping analysis highlighted significant differences between oligometastatic BC patients (12 evaluated globally, due to availability of biological material collected 24 h after RT) and healthy women (*n* = 9) used as control. In particular, before therapy, patients showed reduced percentages of T cells (*p* = 0.045) and increased percentages of NK cells compared to controls (*p* = 0.009; Figure 3). Furthermore, interesting changes, possibly due to RT treatment, were also observed analyzing BC patients before RT and throughout treatment. During treatment, patients maintained significantly higher percentages of NK cells compared to healthy donors (*p* = 0.014 and *p* = 0.015), while T cell fraction displayed a gradual increase that restored its percentage to normal levels (at 1 month after SBRT) (Figure 3). We also noticed a significant decrease in the percentage of B cells 1 month after RT (*p* = 0.019; Figure 3). CD4^+^ T cells were significantly more activated (CD69^+^) in patients compared to controls, especially after RT (*p* = 0.046; Figure 3), while CD8^+^ T cells did not show improved activation globally (not shown). The analysis of immune cells involved in the regulation of antitumor T cell response disclosed higher percentages of myeloid-derived suppressor cells (MDSC) in patient samples, before and after RT, if compared to donors, in particular when the CD14^+^ HLA-DR^−^ SSCim subset (*p* = 0.001 for “before” and “24 h after SBRT,” *p* = 0.01 for “1 month after SBRT”; Figure 3 MDSC4) is considered. We also registered an increased percentage of Treg, 1 month after RT (*p* = 0.046 compared to “before SBRT”; *p* = 0.024 compared to “donors”), and of T helper 17 cells, before and after treatment, in BC patients compared to donors (Figure 3; *p* = 0.001 for “before SBRT,” *p* = 0.003 for “after SBRT”).

### Radiation-Induced Immunomodulation on *In Vitro* BC Models

Three BC cell lines, representative of different BC subtypes, were γ-irradiated or treated with one or three daily doses of 10 Gy (or one single dose of 30 Gy) from 6 MV photon beams, to investigate the direct immunomodulating effects of irradiation on tumor cells.

After 72 h, no significant difference was observed in cell count comparing 10 Gy treated cells with those exposed to 30 Gy (both in one fraction through γ-radiation, or with the fractionated RT method). However, enumeration of cells undergoing fractionated RT instead of γ-radiation disclosed a significantly reduced number of MDA-MB231 cells (Table S4 in Supplementary Material).

**Figure 4:**
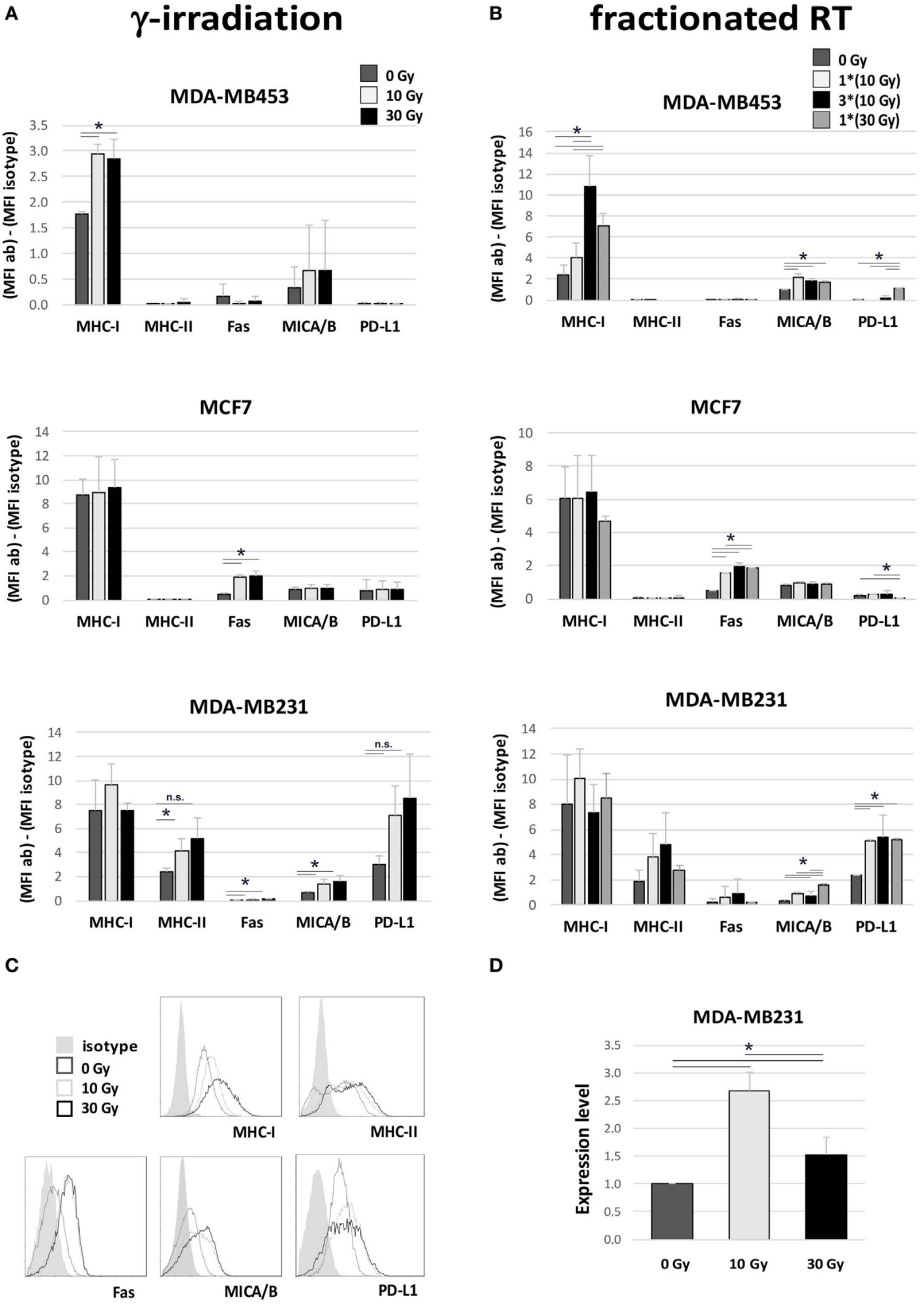
Immunomodulating effects in breast cancer (BC) *in vitro* models after irradiation. Flow cytometry **(A–C)** and gene expression **(D)** analyses performed in three different BC *in vitro* models to evaluate variations in the expression of proteins involved in the interplay between tumor cells and immune effectors, 72 h after irradiation. **(A,B)** Difference in mean fluorescence intensity (MFI) between target-specific antibody and isotype control in untreated sample (dark gray, 0 Gy), sample irradiated with 10 Gy (light gray), sample irradiated with 30 Gy **(A)** or three daily fractions of 10 Gy **(B)** (dark), and sample irradiated with 30 Gy through MV photon beams (gray). Data are represented as mean and SD of three independent experiments. MICA/B, MHC class I polypeptide-related sequence A and B; PD-L1, programed death ligand-1. **(C)** Exemplary histogram plots of flow cytometry analysis. Full curve, isotype control; dark gray curve, untreated sample (0 Gy); light gray curve, sample treated with 10 Gy; black curve, sample treated with 30 Gy. **(D)** Gene expression analysis of *indoleamine 2,3 dioxygenase* (*IDO*) in MDA-MB231 untreated (dark gray), or irradiated with 10 Gy (light gray) or 30 Gy (dark), performed by quantitative real time PCR. Data are represented as mean and SD of expression level measured in three independent experiments (*p ≤ 0.05).

We further monitored the expression of several surface molecules involved in the interplay between tumor cells and immune cells. Significantly increased expression of MHC-I was observed in MDA-MB453 cells after γ-radiation (10 and 30 Gy), and after fractionated RT (only 30 Gy, both fractionated and single dose), while MHC-II was enhanced in MDA-MB231 after γ-irradiation (*p* = 0.05 for 10 Gy, *p* = 0.06 for 30 Gy) (Figures 4A,B). Fas was upregulated in MCF7 with both irradiation methods (after 10 and 30 Gy) and also in MDA-MB231 cells after γ-radiation (10 and 30 Gy) (Figures 4A,B). An increase in the major histocompatibility complex class I-related chain A/B (MICA/B) was highlighted in MDA-MB453 cells undergoing fractionated RT (10 and 30 Gy) and in MDA-MB231 cells γ-irradiated (10 and 30 Gy), or treated with only one dose of fractionated RT (10 and 30 Gy in single dose; Figures 4A,B).

In addition to these potential immunogenic effects, we also noticed increased expression of molecules involved in immune-suppressive mechanisms. In particular, we found an upregulation of PD-L1 in MDA-MB231 cells after fractionated RT (*p* < 0.05; Figure 4B) and, to a lesser extent, also after γ-radiation (Figure 4A), and in MDA-MB453 after a single dose of fractionated RT (30 Gy; Figure 4B). Moreover, real time qRT-PCR analysis also revealed significantly increased levels of *IDO*, an enzyme inducing an immunosuppressive microenvironment, in MDA-MB231 cells, particularly after irradiation with 10 Gy (*p* < 0.05; Figure 4D).

### High Levels of Inflammatory Cytokines in Patients’ Serum and in RT-Treated Cell Lines Supernatant

**Figure 5:**
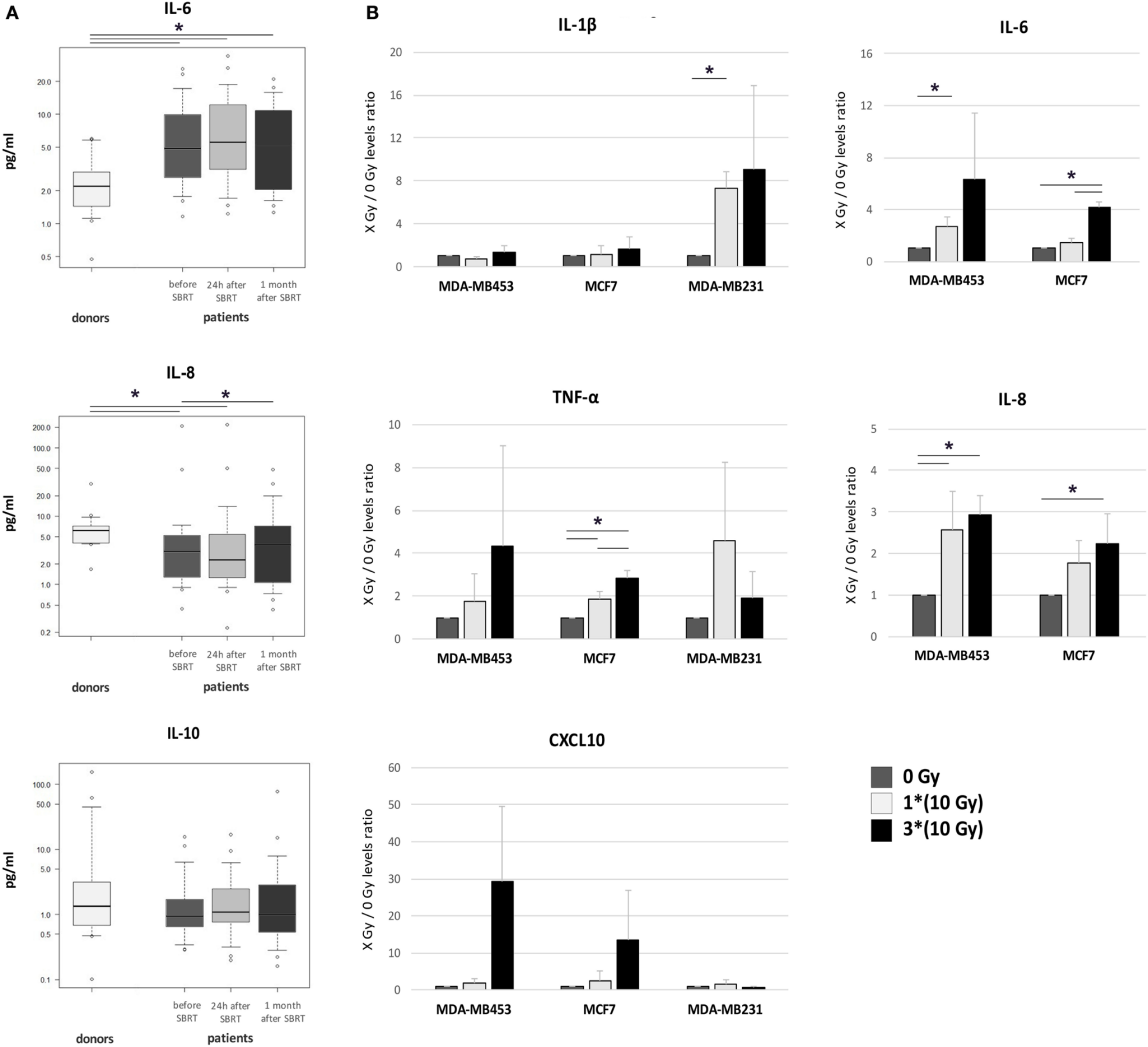
Cytokine levels in patient serum and in the supernatant of irradiated breast cancer (BC) *in vitro* models. **(A)** Serum levels of IL-6, IL-8, and IL-10 were monitored in healthy women (donors, *n* = 14) and in oligometastatic BC patients (*n* = 21) before SBRT (dark gray), 24 h after the first SBRT fraction (light gray), and 1 month after SBRT (dark), through multiplex ELISA arrays. The values in the *Y* axis are reported in a logarithmic scale. **(B)** The amount of cytokines and chemokines released by BC *in vitro* models after irradiation was monitored in culture supernatant collected 72 h after treatment, through multiplex ELISA arrays. Data are represented as mean and SD of the ratios between treated (one SBRT fraction of 10 Gy, light gray; three SBRT fractions of 10 Gy, dark) and untreated samples (dark gray) measured in three independent experiments. IL-6 and IL-8 levels in MDA-MB231 were above the detection limit in all conditions (*p ≤ 0.05).

Serum levels of five different cytokines were evaluated before SBRT, 24 h after the first irradiation (10 Gy) and 1 month after RT in all 21 oligometastatic BC patients and in 14 healthy women used as controls. Patients showed increased levels of the IL-6 pro-inflammatory cytokine at all time points, if compared to healthy donors (*p* < 0.05; Figure 5A). Serum amount of the IL-8 chemokine was lower in patients compared to controls at baseline (*p* < 0.05), whereas it significantly augmented 1 month after SBRT (*p* < 0.05), reaching levels similar to those of controls (Figure 5A). No differences were found in the levels of the anti-inflammatory cytokine IL-10 (Figure 5A), while the levels of IL-1β and TNF-α were undetectable.

Pro-inflammatory cytokines were quantified also in the supernatant of BC cell lines 72 h after irradiation with one or three daily doses of 10 Gy from 6 MV photon beams. Notably, we found increased amounts of IL-1β after 10 Gy treatment in MDA-MB231 cells (*p* < 0.05; Figure 5B). Under standard culture condition, IL-6 supernatant levels in MDA-MB231 cells were too high to evaluate further increases (data not shown), while significant IL-6 amounts were already detectable after the first RT dose (10 Gy) in MDA-MB453 cells (Figure 5B; *p* < 0.05), and after three RT doses (30 Gy) in MCF7 cells (*p* < 0.05; Figure 5B). IL-8 showed enhanced levels after 10 and 30 Gy in MDA-MB453 cells, and after 30 Gy in MCF7 cells (Figure 5B; *p* < 0.05), while it was found barely above the detection limit in MDA-MB231 supernatants (data not shown). Finally, TNF-α increased after 10 and 30 Gy in the culture medium of MCF7 cells (Figure 5B; *p* < 0.05).

We also observed increased amounts of the CXCL10 chemokine (even if with high variability among experiments; *p*-value not significant; Figure 5B), able to recruit immune effectors as T lymphocytes, NK cells, and dendritic cells in the tumor microenvironment, in MDA-MB453 and MCF7 models after three doses of RT. No differences were observed in CXCL16 levels, whereas IL-10 and CXCL9 were not detectable in the culture medium of the BC cell lines investigated.

### SBRT Influence on the Interplay between T Cells and BC Cells

**Figure 6:**
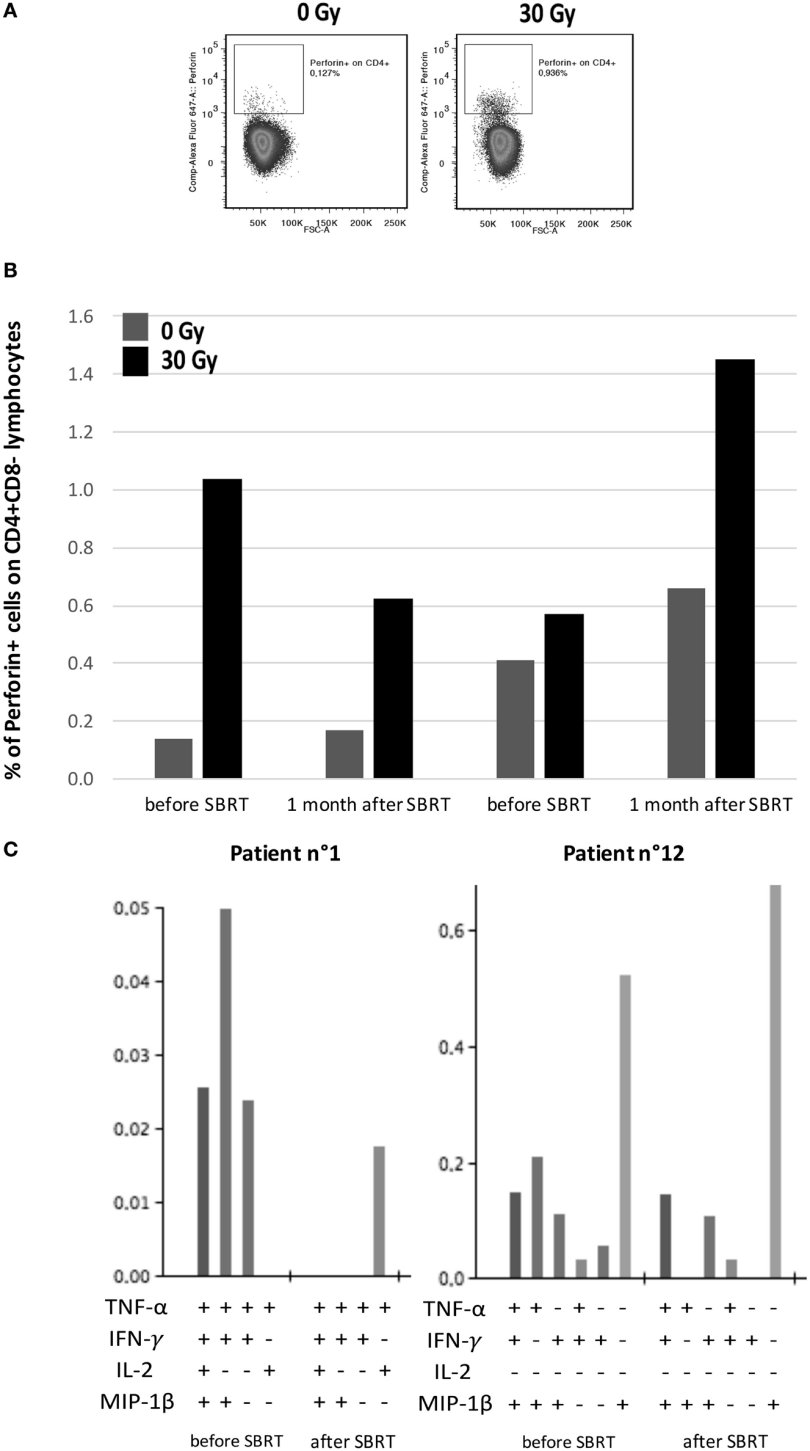
Interplay between patients’ tumor-specific T cells and irradiated breast cancer *in vitro* model. **(A)** Representative flow cytometry dot plots showing perforin^+^/CD4^+^ lymphocytes in intracellular staining after 12 h of co-culture with MDA-MB231 untreated (left) or treated with 30 Gy (right) 72 h before. **(B)** Percentages of perforin^+^ cells among CD4^+^ lymphocytes collected from patients no. 1 and no. 12 before SBRT or 1 month after SBRT and co-cultured with MDA-MB231 untreated (dark gray) or irradiated with 30 Gy (dark) 72 h before, at an effector:target ratio of 2:1. **(C)** Histogram of Boolean analysis performed in patients no. 1 and no. 12 and showing polyfunctional T cells after stimulation with MHC class-II-restricted survivin-derived peptides, before and after SBRT. Each bar represents a population of T cells producing a distinct set of cytokines as reported below.

SBRT-induced upregulation of MHC molecules in *in vitro* BC models, together with the boosting of T cell responses in oligometastatic BC patients after SBRT, prompted us to evaluate whether PBMCs collected from patients before and after SBRT exerted different ability to recognize untreated or irradiated BC cell lines. We thus co-cultured the MDA-MB231 cell line, which showed enhanced MHC class II expression after SBRT (Figure 4), with PBMCs collected from two HLA class II-matched patients (patients no. 1 and no. 12; Table S1 in Supplementary Material) showing CD4^+^ T cell responses against survivin (Figure 1). Interestingly, we observed an enhanced production of perforin by CD4^+^ T cells when PBMCs were cultured in the presence of irradiated BC cell line, compared to untreated targets (Figures 6A,B). Moreover, the percentage of CD4^+^ T cells with cytotoxic potential (perforin^+^) was strictly related to the prevalence of polyfunctional survivin-specific CD4^+^ T cells, before and after SBRT (Figures 6B,C).

### Correlation of Immune Parameters with PFS

Finally, we wondered whether the immune parameters investigated could correlate with PFS. For correlative analysis, patients’ population was divided into two groups for each immune parameter, by using the marker baseline median value as cutoff point and identifying “high” and “low” subgroups.

**Figure 7:**
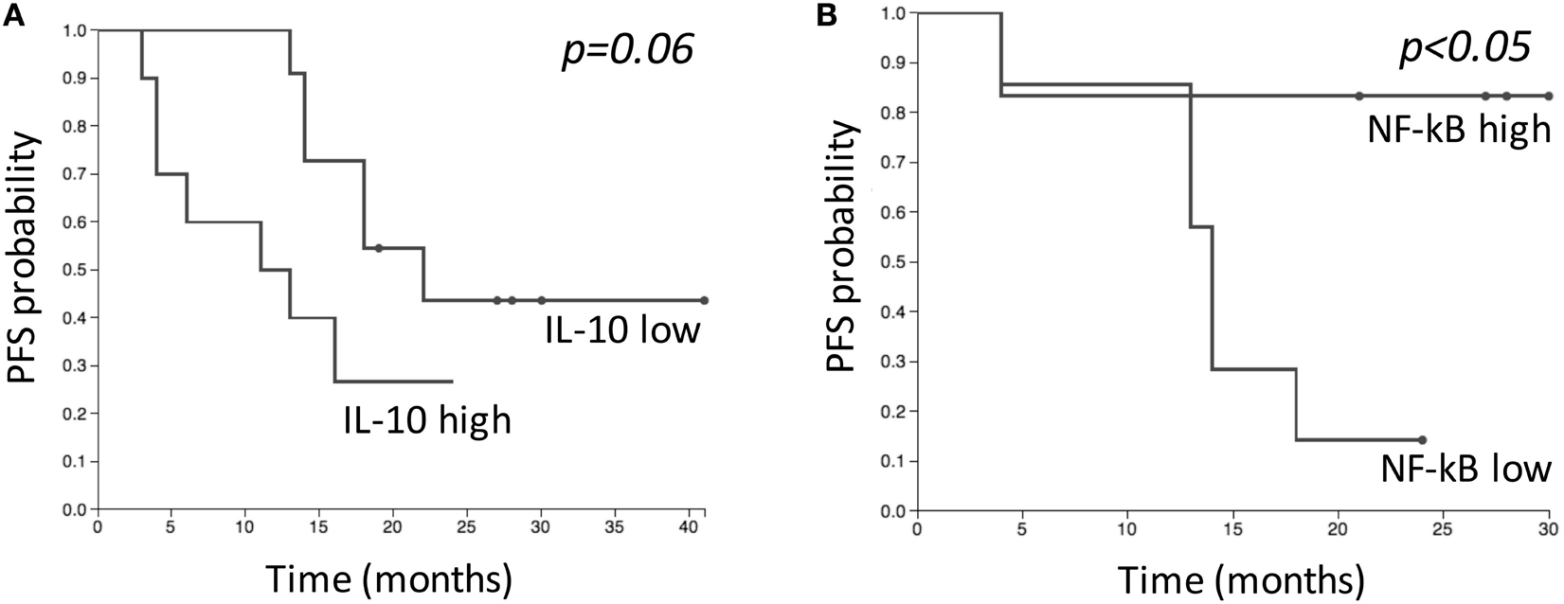
Kaplan–Meier estimates comparing progression-free survival (PFS) of patients with “low” and “high” levels of IL-10 **(A)**, and NF-κB in natural killer cells **(B)** before SBRT. “Low” and “high” groups were defined based on median baseline value of each parameter. **(A)** “Low” group (*n* = 11); “high” group (*n* = 10). **(B)** “Low” group (*n* = 7); “high” group (*n* = 7).

IL-10 amounts and NF-κB levels in NK cells were correlated with PFS. Two-year PFS for patients with “low” (*n* = 11) and “high” (*n* = 10) IL-10 levels was 44 and 27%, respectively (*p* = 0.06) (Figure 7A). Two-year PFS for patients with “low” (*n* = 7) and “high” (*n* = 7) NF-κB levels in NK cells was 14 and 83%, respectively (*p* = 0.03) (Figure 7B). No differences were observed regarding PFS and NK cell numbers. The analysis performed on baseline levels of IL-6, IL-8, B, T, Treg, and Th17 cells did not highlight any correlation between low and high levels of immune parameters and PFS.

## Discussion

This study focused on oligometastatic BC patients included in a clinical trial enrolling a larger cohort of patients compared to those described in the present paper, and demonstrating that high doses of RT (administered through SBRT or intensity modulated RT) are able to achieve local control, which translates into a PFS advantage (23). At 2 years, more than half of treated patients were free from local and distant progression, thus showing that oligometastatic disease is well controlled and potentially curable with local therapies. The results reported in the present study support the immunogenic potential of SBRT in this advanced disease, highlighting significant changes on antitumor immunity not only locally but also systemically. Indeed, SBRT appeared to “switch-on” antitumor immune responses in a sizeable fraction of patients, potentially acting synergistically with immunomodulating drugs used in this setting, such as trastuzumab. However, we also noticed the induction of counteracting immunosuppressive mechanisms potentially able to limit the immunogenic properties of SBRT.

We globally found that the percentage of circulating T cells, reduced before treatment with respect to healthy controls, were restored after SBRT, thus warranting the potential reservoir of antitumor T cells. Conversely, B cell percentages were significantly reduced 1 month after RT, probably due to their radiosensitivity, which is known to be the highest among PBMCs (24). A study comparing the effects of RT with the consequences of RT after adjuvant chemotherapy on the composition of lymphocyte subpopulation in a cohort of BC patients, revealed that the B cell drop is a peculiarity of RT alone (25). We must underline that our phenotype analysis was performed in a limited number of patients and only in a fraction of patients belonging to the clinical trial, due to availability of biological material, thus we cannot conclude that B cells decrease strictly depends on SBRT treatment. To verify whether these effects are specifically related to SBRT treatment in our BC background, we compared the fluctuations observed in this study with previous analysis performed on patients affected by locally advanced BC and treated with neoadjuvant chemotherapy (Table S5 in Supplementary Material) (15, 16). We noticed that both groups of patients showed reduced B cell numbers, probably as a collateral effect of therapy, and increased T cells percentages, as a possible consequence of the concomitant reduction of the B cell compartment. Our detailed analysis revealed improved T-cell responses against BC-associated antigens in a third of patients, supporting the immunogenicity of this RT modality (9). Interestingly, in 3 cases, we observed even a *de novo* appearance of survivin- or mammaglobin-A-specific T-cell responses, suggesting that SBRT may actually stimulate the immune system acting as a cancer vaccine (8). These findings indicate that the presence of spontaneous antitumor T-cell responses already at diagnosis does not represent a mandatory condition to induce a boost in antitumor immunity. Notably, we also observed that, after SBRT, BC antigen-specific T cells produced increased levels of cytokines and a higher number of cytokines per cell, consistent with the induction of multifunctional antitumor T-cell responses of potential therapeutic relevance (26).

Increased numbers of tumor infiltrating lymphocytes (TILs) expressing cytotoxic granules were documented after irradiation (27), and high prevalence of TILs is usually associated with better outcome for BC patients (14). After SBRT in particular, increased numbers of Ki-67^+^ proliferating CD8^+^ T cells were documented both in tumor and at the tumor-stromal interface compared to pre-treatment specimens (28). Unfortunately, we could not evaluate tumor infiltrating lymphocyte after SBRT since we obtained local control in all irradiated lesions and no local recurrence was documented, thus no biopsy was feasible on *in vivo* irradiated tumors. Several factors may contribute to the induction of TILs upon irradiation, particularly the increased local expression of cognate peptide/MHC-I complexes (27). Our *in vitro* cellular models treated with the same SBRT dose used in patients showed increased expression of both MHC-class I (in MDA-MB453 cells) and MHC-class II (in MDA-MB231 cells) molecules, probably favoring the infiltration and recognition by CD8 and CD4 T cells, respectively. Moreover, in the peripheral blood of some oligometastatic patients, we observed increased percentages of both CD4^+^ and CD8^+^ T cells specific for BC antigenic epitopes. Intriguingly, in the global cohort of patients, compared to controls, after treatment we observed an increased percentage of activated CD4^+^ T cells that could recirculate to multiple metastatic sites and potentially mediate abscopal effects (29). Moreover, the higher amounts of the CXCL10 chemokine released by irradiated BC cells *in vitro* may further contribute to the tumor infiltration by T cells observed after SBRT (30).

*In vitro* irradiation of BC cell lines also revealed an increased release of TNF-α and IL-1β pro-inflammatory cytokines. The release of IL-1β, which may contribute to T-cell priming by dendritic cells in tumor microenvironment (7), has been associated with the production of other immunomodulatory molecules, such as HMGB1 and ATP, from radiation-exposed tumor cells that favor antigen processing and cross-presentation by dendritic cells (7). In human BC cells, radiation was also shown to induce a significant upregulation of multiple components of the antigen-processing machinery together with calreticulin cell-surface expression, thus favoring the uptake of tumor antigens by dendritic cells and ultimately enhancing specific lysis by cytotoxic T cells (11). In the same study, the authors showed that radiation-induced tumor sensitivity to lysis by cytotoxic T lymphocytes is comparably augmented by single or fractionated doses of radiation, indicating that both regimens may elicit effective antitumor immune responses (11). In our models, we observed a lower cell viability after fractionated SBRT, compared to a single dose of irradiation, even though both methods induced modulation of surface molecules involved in the interplay between immune effectors and tumor cells. The different radiation types used in the present study seemed to differently modulate the degree of surface expression of some molecules, for example, MICA/B in MDA-MB453 and PD-L1 in MDA-MB231, thus probably indicating that, besides fractionation, radiation type may also have different immunomodulatory activity.

Consistently with these findings, we also provide evidence indicating that irradiated BC cells were better recognized and killed by T cells obtained from patients either before or after SBRT. In particular, culturing patients’ PBMCs with BC cell lines, we interestingly noticed increased percentages of CD4^+^ T cells producing perforin in the presence of irradiated targets compared to untreated cells. Notably, we selected patients showing specific CD4^+^ T-cell responses against survivin, which is expressed by MDA-MB231 cells (31), which in turn was shown to upregulate MHC class-II after γ-radiation. Although preliminary, these results support the fundamental contribution of CD4^+^ T cells in the recognition and killing of tumor cells and their potential boosting induced by SBRT.

In addition to the upregulation of MHC molecules, enhanced expression of the death receptor Fas was observed in MCF7 and in MDA-MB231 cells. Fas triggers a T-cell receptor-independent tumor cell death mechanism, probably exerting thus a potent cytotoxic role if TCR affinity is low (8). MDA-MB453 and MDA-MB231 cells exposed to SBRT also showed upregulation of MICA/B molecules, stress-induced ligands for the NKG2D receptor. NKG2D is mainly expressed by NK cells, γδ T cells, and by a subgroup of αβ CD8^+^ T cells (32). The binding of NKG2D to MICA/B activates cytolytic responses by these cells (33). Interestingly, in half of our patients, we observed an increase in the number of activated NK cells in the blood, measured as nuclear translocation of the p65 subunit of NF-κB shortly (24 h) after the first dose of SBRT. Nuclear translocation of NF-κB in circulating NK cells was associated with increased perforin production and NK cell-mediated cytotoxicity (33). These data thus support the potential contribution of NK cells in mediating the lysis of irradiated tumor cells that may overexpress MICA/B. However, several patients showed a decrease in the number of activated NK cells after SBRT. A larger cohort of patients will allow to determine whether these variations may correlate with patients’ outcome. Although confirmation in larger series is warranted, our results also showed a parallel, nearly significant correlation between *in vitro* ADCC efficiency and p65 nuclear translocation, thus suggesting that the improvement in systemic NK cell activation may favor NK cell-dependent mechanisms, particularly trastuzumab-mediated ADCC in the HER2^+^ subset (22). Consistently, in a different cohort of BC patients, we recently obtained evidence supporting the possible association between improved NK cells activity and the induction of a pathological complete response during neoadjuvant treatment with trastuzumab and paclitaxel (16). Accordingly, here we reported a higher survival free from disease progression in those patients displaying higher percentages of activated NK cells before SBRT, further supporting the potential predictive role of this analysis.

We also found that patients characterized by low IL-10 serum levels before treatment showed a higher PFS, after SBRT, compared to patients with high levels of this cytokine at baseline. IL-10 is an anti-inflammatory cytokine with pleiotropic functions, able to exert both antitumor and tumor-promoting effects. Since high levels of IL-10 were found in metastatic BC patients, it was proposed that higher serum amounts of this cytokine may contribute to immune surveillance impairment, favoring tumor development and progression (34). Thus, the association between increased baseline levels of IL-10 and reduced PFS after SBRT, as observed in the present study, may be an indicator of an underlying systemic immune suppression that could hamper the full exploitation of the immunogenic potential of RT and therefore prevent the prolongation of PFS.

When comparing biological effects induced by SBRT in oligometastatic BC patients and in *in vitro* models of BC, we observed augmented levels of IL-8 in both patients’ serum and BC cells’ supernatant, thus suggesting that this cytokine may contribute to the local inflammatory response induced by SBRT (35). This fluctuation seemed to be specific of RT treatment, since we did not observe the same modulation after chemotherapy (Table S5 in Supplementary Material) (15). In particular, in BC patients, a lower IL-8 expression in immunohistochemistry after RT was associated with local recurrence (36), while after SBRT, we observed increased and restored IL-8 levels and local control was documented in all patients. Furthermore, at diagnosis, oligometastatic BC patients showed higher levels of serum IL-6 compared to healthy donors, and maintained this difference also after SBRT. IL-6 overexpression was described in several malignancies and was associated with advanced disease and tumor progression (37). In our *in vitro* BC models, we observed increased IL-6 release after RT suggesting that SBRT may favor the induction of this endogenous pyrogen, responsible of inflammatory response (10). Compared to healthy women, BC patients showed also higher percentages of circulating inflammatory T helper 17 cells, whose differentiation is promoted by IL-6 and may be sustained by the inflammatory condition induced by SBRT (38), even if we did not observe significant changes in these cells, as instead we had noticed after chemotherapy (Table S5 in Supplementary Material) (16). On the other hand, evidence has been provided suggesting that the inflammatory response induced by IL-6 may inhibit antitumor immune responses (39), thus implying, in our setting, possible immunosuppressive consequences after SBRT due to increased IL-6 release. Indeed, in BC patients, we noticed increased numbers of Treg cells after treatment and globally higher levels of MDSC compared to healthy donors. Also in this case, we must highlight the limited number of patients investigated in the present paper that does not allow us to definitely assert that Treg enhancement is dependent on SBRT treatment. The expansion of these cell populations has been correlated with advanced disease in cancer patients (40, 41) and, notably, both Treg and cells of myeloid-origin appeared much more radioresistant compared to other immune cells (24). We had noticed increased Treg percentages also after chemotherapy (Table S5 in Supplementary Material) (16). However, previous evidence showed that Treg increase seemed to be more evident after RT alone compared to RT plus chemotherapy (25, 42). These findings are consistent with the notion that, in addition to its immunogenic activity, RT triggers also homeostatic immunosuppressive mechanisms that may counterbalance antitumor effects (43). These latter usually predominate, but often they are still not strong enough to sufficiently shift the balance in favor of immunogenicity to promote tumor rejection (43).

Accordingly, *in vitro* experiments revealed a clear induction of immunosuppressive pathways and highlighted enhanced expression of the immune checkpoint molecule PD-L1 and the enzyme *IDO* in BC cell lines after irradiation. PD-L1 upregulation was dose dependent, showing the highest levels at 30 Gy, while *IDO* was more strongly upregulated after a single dose of 10 Gy, thus suggesting that the activation of the PD-1/PD-L1 pathway is probably one of the main mechanisms involved in SBRT-induced immunosuppression. While in our *in vitro* experiments the induction of both these molecules by RT was solely dependent on tumor cells, *IDO* and PD-L1 upregulation occurring *in vivo* may be also a physiological response to IFN-γ production within the tumor microenvironment (44, 45). Constitutive *IDO* expression in cancer is closely related to an autocrine IL-6 signaling loop (46), which could be operational also in patients treated with SBRT, as suggested by enhanced IL-6 release after treatment. Interestingly, PD-L1 upregulation was reported after irradiation of other *in vitro* cancer models, using different fractionated schedules, and employing at most 10 Gy (45). Notably, this molecule and its receptor (PD-1) have become target of currently available drugs as avelumab (anti-PD-L1) and nivolumab (anti-PD-1) that could have a potential synergy with RT. Indeed, a higher frequency of abscopal effects was observed after a combination of immunotherapy and RT, in comparison to RT alone (47, 48). In particular, abscopal responses were documented in several melanoma patients when RT was associated with α-CTLA4 drugs as ipilimumab (49, 50), and/or α-PD-1/PD-L1 antibodies as nivolumab and pembrolizumab (51, 52). The ability to target immunostimulatory and inhibitory checkpoints through monoclonal antibodies could clear the ground for the immunogenic effects, locally induced by RT, and may promote their consequence also systemically (8). On the other hand, other promising immunotherapeutic approaches as cancer vaccines can exploit the immunostimulatory environment created by RT to achieve effective antitumor immune responses (11). Therefore, the combination of a local therapy, such as RT, able to promote an *in situ* immune priming, together with systemic immune modulating agents, such as immune checkpoint inhibitors, cytokines, or cancer vaccines, may synergistically contribute to enhance tumor immunogenicity potentially improving abscopal effects and clinical responses (7).

Several questions regarding the best therapeutic schedule that combines RT with immunotherapy, such as timing, sequence, and dose and technique of radiation, are still unsolved (53). An interesting issue is the site of the irradiated tumor lesion and whether this may influence the probability to observe abscopal responses (53). In our case study, the highest degree of antitumor T-cell induction was observed in the only case showing a metastatic lung lesion (patient no. 9), while the other sites were bone and lymph nodes (Table 1). A recent paper by Poleszczuk et al. proposed a mathematical model that estimates the distribution of focal therapy-activated T cells between metastatic lesions, demonstrating that the distribution of antitumor effectors varies significantly among different metastatic sites and depends on the immune system activation site. They suggested thus that the choice of the metastases to irradiate could influence the probability to induce systemic metastatic regression (29).

Finally, the different immunomodulatory effects induced by SBRT may also depend on tumor heterogeneity, as suggested by the different variations in protein expression observed among BC cell lines. Indeed, BC is characterized by a complex biological heterogeneity, which may respond differently to the immunomodulating effects induced by SBRT. The genetic alterations causing dysregulated cellular pathways that characterize each BC subtype may have different effects on tumor cell immunogenicity (54). Similarly, the different behavior of BC subtypes to irradiation may differently favor the induction of immunogenic effects and shift the balance in favor of antitumor effects. A main limitation of the present study is the relative low number of enrolled patients that did not allow us to stratify cases according to BC subtype. The same analysis performed in a larger cohort of patients will allow a better characterization of the different immunomodulating effects induced by SBRT depending on tumor subtype, to possibly identify those patients who could mostly benefit from this potentially effective treatment modality. The increase in the number of patients could also confirm data obtained in the limited case study of the present paper and finally exclude any possible bias due to missing samples. Another limit of our analysis is the lack of data in *in vivo* models to validate the results obtained *in vitro*. Available data obtained in BC syngeneic mouse models showed that the RT-induced antitumor immunity required a combination with drugs able to counteract immunosuppressive factors (as CTLA4, TGF-β, and PD-1/PD-L1) to improve tumor control by radiation and definitively favor abscopal responses (55–57). This observation may explain why we did not observed any abscopal responses in our case study and stimulate further studies aimed at validating the immune parameters modulated by SBRT as biomarkers potential able to identify those patients who could benefit from a combination of RT with immunotherapy.

In conclusion, our analysis revealed that local SBRT is able to induce systemic effects influencing the antitumor immune response in oligometastatic BC patients. Liquid biopsy may thus represent a useful resource to monitor the potential immunogenic effects of SBRT that in turn could contribute to the curative potential of this treatment not only locally but also systemically. Our data, although preliminary, support that SBRT could induce *in situ* vaccination also in the absence of a pre-existing antitumor immunity, thus supporting the adjuvant role of RT associated with immunotherapy (58). The combined treatment could shift the balance of immunomodulating effects induced by RT in favor of immune-activating signals to finally augment radiation-induced tumor immunogenicity and possibly the curative potential of SBRT in oligometastatic BC.

## Ethics Statement

The study was conducted with the approval of institutional review board of Cro Aviano, National Cancer Institute. Written informed consent was obtained from all patients and donors. All subjects gave written informed consent in accordance with the Declaration of Helsinki.

## Author Contributions

EM conceived the study, performed most of immunological experiments, and drafted the manuscript. DM, EC, and DF performed part of the experiments. AR contributed to sample collection and data acquisition. MA performed cell lines irradiation and contributed to draft the manuscript. CF, MB, LM, AC, and SS collected and analyzed clinical data. RD conceived the study and reviewed the manuscript. MT collected and analyzed clinical data, conceived and designed the study, and reviewed the manuscript. All authors read and approved the final manuscript.

## Conflict of Interest Statement

The authors declare that the research was conducted in the absence of any commercial or financial relationships that could be construed as a potential conflict of interest. The reviewer HE and handling editor declared their shared affiliation.

## Abbreviations

ADCC, antibody-dependent cell cytotoxicity; BC, breast cancer; IDO, indoleamine 2,3 dioxygenase; MICA/B, major histocompatibility complex class I-related chain A/B; MDSC, myeloid-derived suppressor cells; NK, natural killer; OS, overall survival; PFS, progression-free survival; RT, radiotherapy; Treg, regulatory T cells; SBRT, stereotactic body radiotherapy; TAAs, tumor-associated antigens; TILs, tumor-infiltrating lymphocytes.
