# Supplementary material for: Local High-Dose Radiotherapy Induces Systemic Immunomodulating Effects of Potential Therapeutic Relevance in Oligometastatic Breast Cancer
Source: Front Immunol. 2017 Nov 6;8:1476. doi: 10.3389/fimmu.2017.01476 (PMC5681493; doi:10.3389/fimmu.2017.01476)
Supplement: Supplementary file 1 [file presentation_1.pdf]

Local high-dose radiotherapy induces systemic immunomodulating effects of potential therapeutic relevance in oligometastatic breast cancer.

Muraro E[1]*, Furlan C[2], Avanzo M[3], Martorelli D[1], Comaro E[1], Rizzo A[1], Fae' DA[1], Berretta M[4], Militello L[4], Del Conte A[5], Spazzapan S[4], Dolcetti R[6], Trovo' M[7]

*** Correspondence:**
Elena Muraro
emuraro@cro.it

SUPPLEMENTARY TABLES AND FIGURES

Tables

Table S1. Patients' HLA genotyping

Patient number	HLA-class I	HLA-class II
1		DRB1*03
3	A*02	DRB 1*04
4	A*03	DRB 1*01; DRB 1*04
5	A*24; B*35	DRB 1*01
6	A*03; B*35	DRB 1*01; DRB1*03
7	B*35	DRB1*03
8	A*02	DRB1*04
9	A*02	DRB1*01
10	A*03; A*24	DRB1*01; DRB1*03
11	A*24	DRB1*03
12	A*02	DRB1*03
13	A*02; A*24	DRB1*03
15		DRB1*03
16	A*24	DRB1*03
17	A*24; B*35	DRB1*03
19	A*02	DRB1*04
20	A*02; A*24; B*35	DRB1*03
21	A*24	DRB1*03
22	A*02; B*35	DRB1*03
23	A*02; B*35	DRB1*01

Table S2. List of epitopes derived from BC-associated antigens or viral antigens as controls.

BC-associated antigens	HLA allele	Epitope aminoacid position	Epitope sequence	References
Survivin	-A*0201	Survivin 95-104	ELTLGEFLKL	(1)
	-A*0301	Survivin-3a 18-27	RISTFKNWPK	(2)
	-A*2402	Survivin-2b 57-65	AYACNTSTL	(3)
	-B*3501	Survivin 51-59	EPDLAQCFF	(2)
	-DRB1	Survivin 97-111	TLGEFLKLDRERAKN	(4)
Mammaglobin-A	-A*0201	Mammaglobin-A 83-92	LIYDSSLCDL	(5)
	-A*0301	Mammaglobin-A 5-13	PLLENVISK	(6)
	-A*2402	Mammaglobin-A 16-25	CYAGSGCPL	(7)
	-DRB1*0401	Mammaglobin-A 34-48	NPQVSKTEYKELLQE	(8)
Her2/neu	-A*0201	HER-2/neu 347-355	KIFGSLAFL	(9)
	-A*0301	HER-2/neu 754-762	VLRENTSPK	(10)
	-A*2402	HER-2/neu 41-49	TYLPTNASL	(11)
	-DRB1*0401	HER-2/neu 861-877	KVPIKWMALESILRRRF	(12)
**Negative controls**				
HIV	-A*0201	Gag p17 77-85	SLYNTVATL	
	-A*0301	Nef 73–82	QVPLRPMTYK	
	-A*2402	Env gp160 585-593	RYLRDQQLL	
	-B*3501	ARV-2 nef 71-79	FPVRPQVPL	
	-DRB1	p24 164-181	YVDRFYKTLRAEQASQEV	
**Positive controls**				
Flu	-A*0201	Matrix Protein 1 58-66	GILGFVFTL	
CMV	-A*2402	pp65 341-349	QYDPVAALF	
	-B*3501	pp65 123-131	IPSINVHHY	
EBV	-A*0201	BMLF1 280-288	GLCTLVAML	
	-DRB1	EBNA1 514-539	KTSLYNLRRGTALAIPQCRLTPLSRL	

HIV, Human Immunodeficiency Virus; Flu, Influenza Virus; CMV, Cytomegalovirus; EBV, Epstein-Barr Virus.

Table S3. Antibodies for flow cytometry and multispectral imaging.

Antigen	Fluorochrome	Isotype	Clone	Brand
**Lineage Cocktail 2 (CD3, CD14, CD19, CD20, CD56)**	FITC	Mouse IgG1, κ (CD3, CD19, CD20); Mouse IgG2b, κ (CD14, CD56)	SK7(CD3); MφP9(CD14); SJ25C1(CD19); L27(CD20); NCAM16.2(CD56)	BD Bioscience (BD)
**CD3**	FITC	Mouse IgG1, κ	UCHT1	BD
**CD4**	APC-Cy7	Mouse IgG1, κ	RPA-T4	BD
**CD8**	BV421 and Pe-CF594	Mouse IgG1, κ	RPA-T8	BD
**CD11b**	APC-Cy7	Mouse IgG1, κ	ICRF44	BD
**CD14**	BV421	Mouse IgG2b, κ	MφP9	BD
**CD15**	PerCP-Cy5.5	Mouse IgM, κ	HI98	BD
**CD16**	PE-Cy7	Mouse IgG1, κ	3G8	BD
**CD20**	Alexa Fluor 700	Mouse IgG2b, κ	2H7	BD
**CD25 (IL-2Rα)**	APC	Mouse IgG1, κ	M-A251	BD
**CD33**	APC	Mouse IgG1, κ	WM53	BD
**CD45**	BV510	Mouse IgG1, κ	HI30	BD
**CD45RA**	Alexa Fluor 700	Mouse IgG2b, κ	HI100	BD
**CD56**	Pe	Mouse IgG1, κ	B159	BD
**CD69**	BV421	Mouse IgG1, κ	FN50	BD
**CD95 (Fas)**	FITC	Mouse IgG1, κ	DX2	BD
**CD107a (LAMP1)**	FITC and PE	Mouse IgG1, κ	H4A3	BD
**CD124 (IL-4Rα)**	Pe	Mouse IgG1, κ	hIL4R-M57	BD
**CD127 (IL-7Rα)**	PerCP-Cy5.5	Mouse IgG1, κ	HIL-7R-M21	BD
**CD274 (PD-L1)**	PE	Mouse IgG1, κ	MIH1	BD
**CD314 (NKG2d)**	PerCP-Cy5.5	Mouse IgG1, κ	1D11	BD
**CD335 (NKp46)**	APC	Mouse IgG1, κ	9E2/Nkp46	BD
**HLA-ABC**	Pe	Mouse IgG1, κ	G46-2.6	BD
**HLA-DR, DP, DQ**	FITC	Mouse IgG2a, κ	Tu39	BD
**HLA-DR**	PE-Cy7	Mouse IgG2a, κ	G46-6	BD
**MICA/B**	Alexa Fluor 488	Mouse IgG2a, κ	6D4	eBioscience (Affymetrix)
**IL-2**	APC	Rat IgG2a, κ	MQ1-17H12	BD
**IFN-γ**	Pe-Cy7	Mouse IgG1, κ	4S.B3	BD
**TNF-α**	Pe and PerCP-Cy™5.5	Mouse IgG1, κ	MAb11	BD
**MIP-1β**	Pe and Alexa Fluor 700	Mouse IgG1, κ	D21-1351	BD
**Perforin**	Alexa Fluor 647	Mouse IgG2b, κ	δG9	BD
**IL-17A**	Alexa Fluor 647	Mouse IgG1, κ	N49-653	BD
**IL-22**	PerCP-eFluor ®710	Mouse IgG1, κ	22URTI	eBioscience
**Foxp3**	PE	Mouse IgG1, κ	236A/E7	eBioscience
**Ki-67**	PE-Cy™7	Mouse IgG1, κ	B56	BD
**NF-κB p65**	FITC	Mouse IgG1	F-6	Santa Cruz Biotechnology

Table S4. Cell count after irradiation

		γ-irradiation mean±SD	fractionated RT mean±SD	p-value
**MDA-MB453**	*10 Gy*	0.24±0.07	0.26±0.02	0.63
	30 Gy	0.20±0.05	0.23±0.10	0.68
**MCF7**	*10 Gy*	0.20±0.03	0.49±0.13	0.32
	30 Gy	0.23±0.09	0.11±0.06	0.38
**MDA-MB231**	*10 Gy*	0.32±0.07	0.31±0.12	0.88
	30 Gy	0.19±0.02	0.06±0.02	**0.003**

Data are reported as cell count ratio between treated (10 or 30 Gy) and not treated cells 72 hours after irradiation. SD, Standard Deviation; p-value between **γ**-irradiation and fractionated RT.

Table S5. Comparison of immune parameters measured in oligometastatic BC patients treated with SBRT and in BC patients treated with neoadjuvant chemotherapy.

Parameter	SBRT oligometastatic BC	Neoadjuvant chemotherapy in locally advanced BC patients (Miolo et al, 2014; Muraro et al, 2015)
**IL-6**<br>Median value<br>(Min-Max)<br>pg/ml	Higher levels in patients compared to healthy donors<br><br>Patients:<br>A=4.9 (1.2-26.2)<br>B=5.6 (1.2-34.3)<br>C=5.2 (1.3-21.1)<br><br>Healthy donors:<br>2.2 (0.5-6.0)	Increased levels after therapy compared to diagnosis values<br><br>Patients:<br>A=2.0 (0.5-8.5)<br>B=1.3 (0.09-15.7)<br>C=3.3 (0.9-16.3)
**IL-8**<br>Median value<br>(Min-Max)<br>pg/ml	Lower levels in patients compared to healthy donors only at diagnosis, then seemed to improve.<br><br>Patients:<br>A=3.1 (0.4-208.5)<br>B=2.3 (0.2-220.3)<br>C=3.8 (0.4-47.9)<br><br>Healthy donors:<br>6.2 (1.7-29.8)	No changes<br><br>Patients:<br>A=2.3 (0.09-30.3)<br>B=1.7 (0.09-11.1)<br>C=3.7 (0.5-25.3)
**IL-10**<br>Median value<br>(Min-Max)<br>pg/ml	No changes<br><br>Patients:<br>A=0.9 (0.3-15.5)<br>B=1.1 (0.2-16.8)<br>C=1.0 (0.2-78.3)<br><br>Healthy donors:<br>1.3 (0.1-156.4)	Reduced levels after therapy compared to diagnosis values<br><br>Patients:<br>A=0.9 (0.0-10.2)<br>B=1 (0.09-6.3)<br>C=0.5 (0.09-3.5)
**B cells**<br>Mean<br>(Standard Deviation)<br>% on<br>lymphocytes	Reduced levels after SBRT compared to diagnosis levels<br><br>Patients:<br>A=8.9 (5.0)<br>B=8.9 (4.5)<br>C=6.4 (3.8)	Reduced levels after therapy compared to diagnosis values<br><br>Patients:<br>A=8.1 (4.3)<br>B=2.9 (2.2)<br>C=1.4 (1.9)

	Healthy donors:<br>10.0 (4.5)	
**NK cells**<br>Mean<br>(Standard Deviation)<br>% on<br>lymphocytes	Higher levels in patients compared to healthy donors<br><br>Patients:<br>A=15.6 (8.2)<br>B=14.6 (7.3)<br>C=13.4 (6.0)<br><br>Healthy donors:<br>7.4 (4.3)	Reduced levels after therapy compared to diagnosis values<br><br>Patients:<br>A=11.9 (7.2)<br>B=11.7 (7.2)<br>C=9.1 (4.8)
**T cells**<br>Mean<br>(Standard Deviation)<br>% on<br>lymphocytes	Lower levels in patients compared to healthy donors only at diagnosis<br><br>Patients:<br>A=68.3 (8.2)<br>B=70.3 (5.6)<br>C=72.3 (6.4)<br><br>Healthy donors:<br>75.7 (7.4)	Increased levels after therapy compared to diagnosis values<br><br>Patients:<br>A=72.1 (11.2)<br>B=75.0 (11.8)<br>C=80.7 (8.0)
**Treg**<br>Mean<br>(Standard Deviation)<br>% on CD4+ T cells	Increased levels after SBRT compared to diagnosis values and healthy donors<br><br>Patients:<br>A=4.0 (0.8)<br>B=3.8 (0.9)<br>C=4.6 (1.2)<br><br>Healthy donors:<br>3.5 (0.8)	Increased levels after therapy compared to diagnosis values<br><br>Patients:<br>A=2.1 (1.2)<br>B=3.4 (1.2)<br>C=3.6 (1.8)
**Th17**<br>Mean<br>(Standard Deviation)<br>% on CD4+ T cells	Higher levels in patients compared to healthy donors<br><br>Patients:<br>A=0.9 (0.4)<br>B=0.9 (0.8)<br>C=0.9 (0.5)<br><br>Healthy donors:<br>0.3 (0.1)	Improved levels during therapy compared to diagnosis values<br><br>Patients:<br>A=1.1 (1.0)<br>B=2.4 (2.1)<br>C=1.3 (1.1)

A=before therapy; B=immediately after RT/during neoadjuvant chemotherapy (12° week of treatment); C=after treatment.

Figures

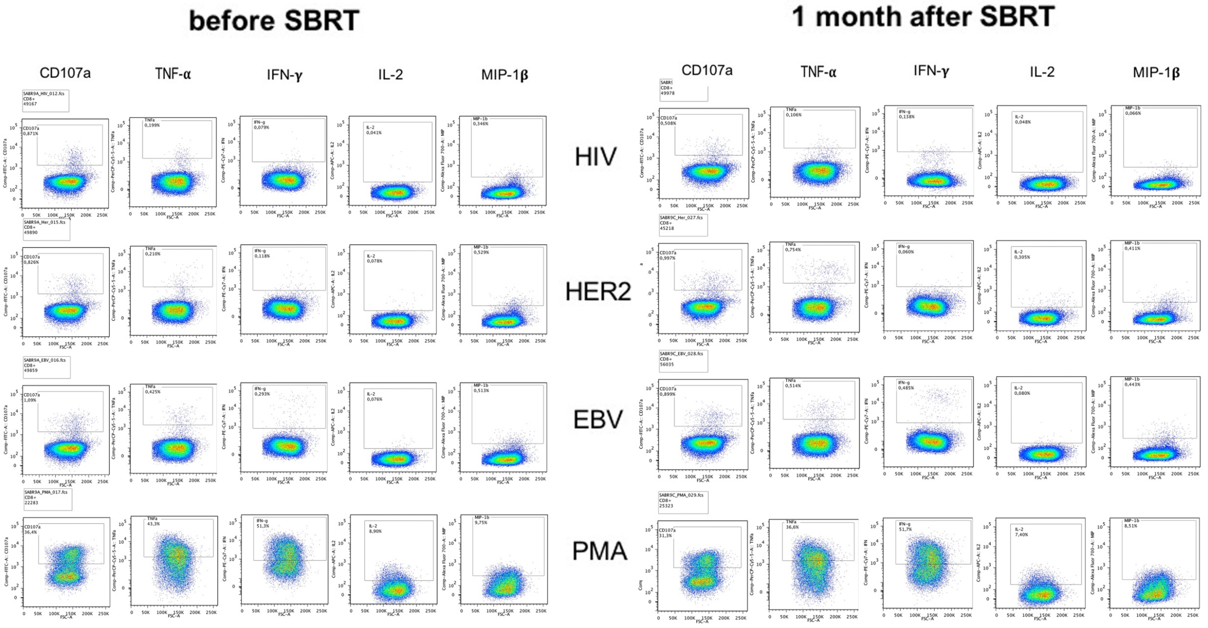

Figure S1. Exemplary dot plot of Intracellular staining analysis performed in flow cytometry. Analysis performed on PBMCs obtained from patients n° 9 before and 1 month after SBRT. Dot plots are gated in $CD4^{-}CD8^{+}$ death marker^{-} cells. The sources of peptides used for stimulation of PBMCs are indicated in the middle. PMA was used as experiment positive control. Each column shows the specific expression of a single cytokine or CD107a. The X axis reports the forward scatter, the Y axis every single cytokine.

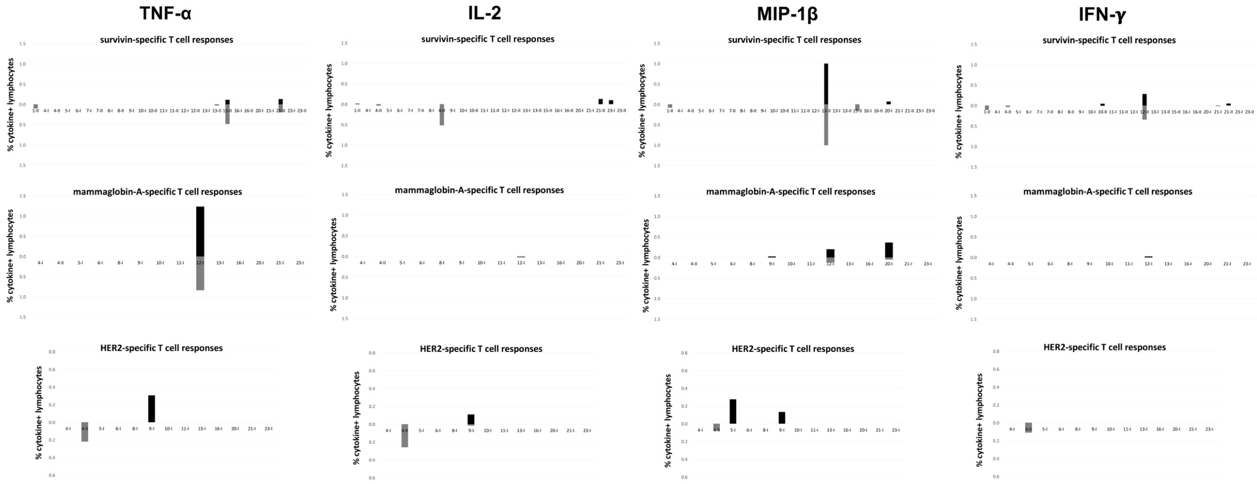

Figure S2. Tumor-specific T cell responses in oligometastatic BC patients before and after SBRT. Graphs showed the percentage of T-cells positive to each of the markers investigated: TNF-α, IL-2, MIP-1β, and IFN-γ. A cut-off of 0.01% was set up to discriminate a positive population. Each pair of histograms (grey, data before SBRT; black, data after SBRT) represents the analysis performed in a single patient, as reported below, after stimulation with MHC class-I (I) or MHC class-II (II) restricted peptides.

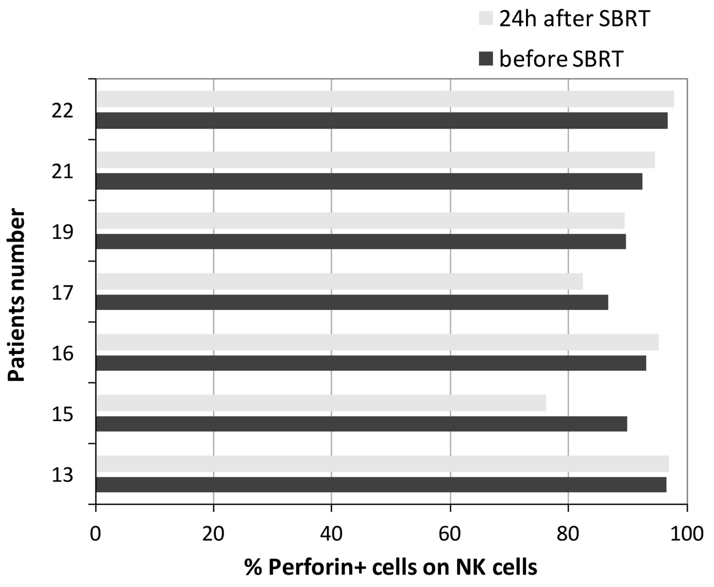

Figure S3. Analysis of Perforin expression in NK cells before and 24 hours after SBRT. Perforin expression was quantified in $CD16^{+}CD56^{+}$ cells through flow cytometry.

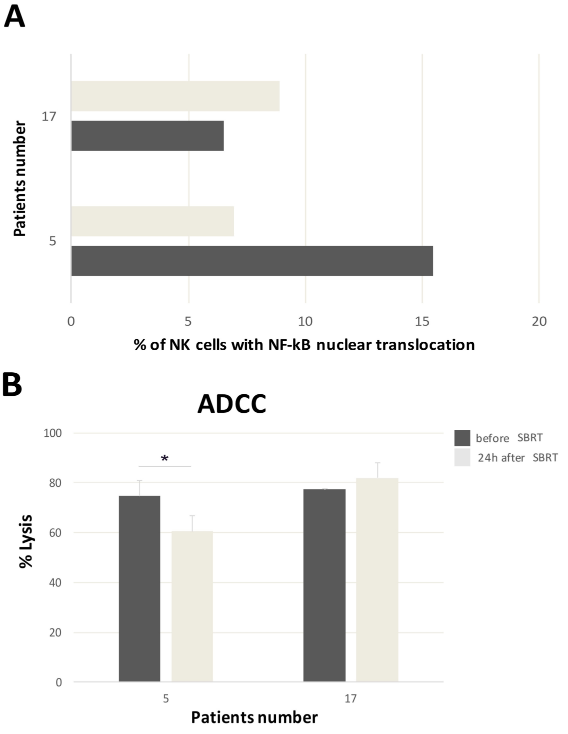

Figure S4. NK cell activation status and ability to mediate ADCC in patients treated with concomitant Trastuzumab. A. Percentage of NK cells showing nuclear translocation of the p65 subunit of the NF-kB complex measured through multispectral flow cytometry, in patients undergoing a concomitant treatment with Trastuzumab (n° 5 and 17) before and 24 hours after the first SBRT fraction. **B.** Histogram representing the amount of target cell (MDA-MB453) lysis induced by PBMCs (Effector:Target ratio 30:1), through ADCC, in the presence of Trastuzumab. Effectors (PBMCs) were collected before (dark grey) or after (light grey) SBRT.

References

1. Schmitz M, Diestelkoetter P, Weigle B, Schmachtenberg F, Stevanovic S, Ockert D, et al. Generation of survivin-specific CD8+ T effector cells by dendritic cells pulsed with protein or selected peptides. *Cancer Res* (2000) 60:4845-9

2. Reker S, Meier A, Holten-Andersen L, Svane IM, Becker JC, thor SP, et al. Identification of novel survivin-derived CTL epitopes. *Cancer Biol Ther* (2004) 3:173-9

3. Hirohashi Y, Torigoe T, Maeda A, Nabeta Y, Kamiguchi K, Sato T, et al. An HLA-A24-restricted cytotoxic T lymphocyte epitope of a tumor-associated protein, survivin. *Clin Cancer Res* (2002) 8:1731-9

4. Widenmeyer M, Griesemann H, Stevanovic S, Feyerabend S, Klein R, Attig S, et al. Promiscuous survivin peptide induces robust CD4+ T-cell responses in the majority of vaccinated cancer patients. *Int J Cancer* (2012) 131:140-9. doi:10.1002/ijc.26365

5. Jaramillo A, Narayanan K, Campbell LG, Benshoff ND, Lybarger L, Hansen TH, et al. Recognition of HLA-A2-restricted mammaglobin-A-derived epitopes by CD8+ cytotoxic T lymphocytes from breast cancer patients. *Breast Cancer Res Treat* (2004) 88:29-41. doi:10.1007/s10549-004-8918-1

6. Jaramillo A, Majumder K, Manna PP, Fleming TP, Doherty G, Dipersio JF, et al. Identification of HLA-A3-restricted CD8+ T cell epitopes derived from mammaglobin-A, a tumor-associated antigen of human breast cancer. *Int J Cancer* (2002) 102:499-506. doi:10.1002/ijc.10736

7. Tiriveedhi V, Sarma NJ, Subramanian V, Fleming TP, Gillanders WE, Mohanakumar T. Identification of HLA-A24-restricted CD8(+) cytotoxic T-cell epitopes derived from mammaglobin-A, a human breast cancer-associated antigen. *Hum Immunol* (2012) 73:11-6. doi:10.1016/j.humimm.2011.10.017

8. Schmidt HH, Ge Y, Hartmann FJ, Conrad H, Klug F, Nittel S, et al. HLA Class II tetramers reveal tissue-specific regulatory T cells that suppress T-cell responses in breast carcinoma patients. *Oncoimmunology* (2013) 2:e24962. doi:10.4161/onci.24962

9. Fisk B, Blevins TL, Wharton JT, Ioannides CG. Identification of an immunodominant peptide of HER-2/neu protooncogene recognized by ovarian tumor-specific cytotoxic T lymphocyte lines. *J Exp Med* (1995) 181:2109-17

10. Kawashima I, Tsai V, Southwood S, Takesako K, Sette A, Celis E. Identification of HLA-A3-restricted cytotoxic T lymphocyte epitopes from carcinoembryonic antigen and HER-2/neu by primary in vitro immunization with peptide-pulsed dendritic cells. *Cancer Res* (1999) 59:431-5

11. Okugawa T, Ikuta Y, Takahashi Y, Obata H, Tanida K, Watanabe M, et al. A novel human HER2-derived peptide homologous to the mouse K(d)-restricted tumor rejection antigen can induce HLA-A24-restricted cytotoxic T lymphocytes in ovarian cancer patients and healthy

individuals. *Eur J Immunol* (2000) 30:3338-46. doi:10.1002/1521-4141(200011)30:11<3338::AID-IMMU3338>3.0.CO;2-3

12. Kobayashi T, Wood M, Song Y, Appella E, Celis E. Defining promiscuous MHC class II helper T-cell epitopes for the HER2/neu tumor antigen. *Cancer Res* (2000) 60:5228-36
